# The use of pasung for people with mental illness: a systematic review and narrative synthesis

**DOI:** 10.1186/s13033-020-00424-0

**Published:** 2020-12-07

**Authors:** Muhamad Taufik Hidayat, Sharon Lawn, Eimear Muir-Cochrane, Candice Oster

**Affiliations:** 1 College of Medicine and Public Health, Flinders University, PO Box 2100, Adelaide, South Australia 5001 Australia; 2 West Java Psychiatric Hospital, Bandung, Indonesia; 3 South Australian Mental Health Commissioner, Adelaide, South Australia Australia; 4 College of Nursing and Health Sciences, Flinders University, PO Box 2100, Adelaide, South Australia 5001 Australia

**Keywords:** Pasung, Lived experience, Systematic review, Narrative synthesis, Community mental health, Restraint, Human rights

## Abstract

**Background:**

Pasung is the term used in Indonesia and a number of other countries for seclusion and restraint of people with mental illness in the community, usually at home by their family. While pasung has been banned because it is contrary to human rights, its practice continues to exist within the community, particularly where community mental health services are limited, and in the absence of adequate social support, and pervasive negatives beliefs about mental illness. It is essential to understand the reasons for the ongoing use of pasung and to examine potential solutions.

**Methods:**

A systematic review and narrative synthesis of peer-reviewed international literature was conducted to identify the socio-cultural contexts for pasung use, and interventions to address it. The analysis draws on the socio-ecological framework, which focused on relationships between the individual and their environment.

**Result:**

Fifty published articles were included in the review; all studies were conducted in Asia and Africa, with 32 undertaken in Indonesia. Most studies were qualitative (n = 21). Others included one case–control study, one cross-sectional study, and seven surveys; only four studies examined the application of an intervention, and each used a pre and post methodology. Of these, two studies tested psychoeducational interventions which aimed to overcome family burden due to pasung, and each suggested a community mental health approach. The remaining two studies evaluated the intervention of ‘unlocking’; one study used a community-based culturally sensitive approach, and the other used a community-based rehabilitation program. Reasons for pasung given by family appear to be as a last resort and in the absence of other supports to help them care for the person with severe mental illness.

**Conclusion:**

The findings highlight that a mixture of individual, interpersonal, community and policy interventions are needed to reduce the use of pasung. While consumer and carer involvement as part of a socio-ecological approach is understood to be effective in reducing pasung, an understanding of how to elaborate this in the management of pasung remains elusive.

*Review Registration* CRD42020157543: CRD

## Introduction

Seclusion and restraint are interventions used to control people with mental disorders (PWMD) who exhibit aggression and violence in psychiatric settings [1]. However, little is known about their use in non-psychiatric settings, particularly in a community environment such as a home. These community confinement methods are a global phenomenon and are found in many countries. However, the practice is most commonly found in low- and middle-income countries where mental health services are under-resourced [2–5]. In Indonesia, this practice is called pasung, which entails the physical restraint, confinement, stock or shackling of the person in the community, often inside their homes. Usually, pasung use is related to and applied to people who are considered to be severely mentally ill and a danger to themselves or other people [3, 6].

Pasung uses one or more combinations of different methods including mechanical restraint and isolation/seclusion. Mechanical restraint in pasung usually involves the use of chain shackles, rope, or wooden stocks. Isolation is typically comprised of locking people in confined cages and hidden spaces located at a distance from the community or in a separate hut [7–9]. Both of these processes can be experienced concurrently; that is, the person can be chained and confined at the same time. Most of these places are dirty, with the PWMD sometimes defecating and urinating without access to a toilet, with no ventilation aside from a small window to insert food and extremely limited human contact. Limited space means the PWMD needs to sit or lie down on the floor for the duration of their confinement. As a result, many of those subjected to pasung have been found to be undernourished, physically wasting and ill from a range of untreated health conditions, and sometimes deceased [6, 10].

Although pasung has been officially banned in Indonesia since 1977, its practice has persisted and continued to rise [11]. This led to the establishment in 2010 of the Free Pasung Program in Indonesia (Indonesia Free from Restraint and Seclusion), which aimed to reduce the use of community restraint for PWMD. Furthermore, in 2011, to ensure equal rights for PWMD and that they were not subjected to torture and mistreatment, the government ratified the Internationally recognised Convention on the Rights of Persons with Disabilities (CRPD) [12]. It was followed by the ratification of the Mental Health Act no.18, enacted since 2014, which also re-affirmed that the perpetrators of deprivation of others would be imprisoned and fined [13].

Until recently, there were no epidemiological surveys that accurately conveyed the percentage of people being restrained in the community in Indonesia [3, 14]. The Ministry of Health of Indonesia estimated that there had been approximately 57,000 PWMD subjected to pasung at least once in their lifetime in 2014 and 18,880 currently in pasung [15]. However, the number of PWMD in pasung might be far more than the number predicted since the majority of PWMD are hidden by their family and not exposed to public view. Indonesian culture and society generally perceive mental disorder as shameful [6, 16].

The Indonesian government claims that the Free Pasung Program has successfully reduced rates of pasung from 18,880 cases to 12,220 in 2018 [11]. Repeated revisions of the program by the Indonesian Government (2010–2017 and 2019) have sparked doubt about the actual number of people still in pasung. In addition, data released by the Indonesian Institute of Health Research showed a slight decrease from 14.3 to 14% of PWMD who were subjected to pasung, but this does not match the reduction in rates cited by the Ministry of Health [15]. Nor does it appear to account for the increase in population growth in Indonesia during the period 2010–2018 of almost 30 million (from 237 to 265 million) [17]. Other data from the Ministry of Health showed 10% of those who were in pasung were released and treated in hospital over the 6-year period from 2009 to 2014. However, there is no data on how many of those PWMD were successfully supported through rehabilitation or returned to pasung in the community.

The continued practice of pasung indicates that there are likely complex issues present within Indonesian society that contribute to its continued use, and that a more comprehensive understanding and solution is needed [3, 4]. International evidence has confirmed that PWMD are not predominantly those who commit violence but instead are more likely to be victims of violence perpetrated by others [3, 4, 18]. Examining the use of pasung is essential to address fundamental human rights violations associated with pasung [19, 20].

This systematic review aimed to: (i) explore the use of pasung in community settings, the experiences of those involved, and the perceived reasons for its use; and (ii) explore current interventions and potential solutions to pasung. For the purposes of this review, the definition of pasung is limited to the physical restraint, confinement, stock or shackling of the person in the community. The research questions were:

1. What is the nature of pasung, and the reasons given by families and communities for its use?
2. What programs (formal, informal and innovations) have been implemented to overcome the practice of pasung, and what is the effectiveness of these programs?

## Methods

### Search strategy

The following search strategies were undertaken in September 2019 to locate peer reviewed articles with the search term ‘pasung’, developed in consultation with an expert librarian. The search involved the following databases: MEDLINE, PsycINFO, CINAHL, Scopus, ProQuest, Ovid Emcare, Google Scholar. We constructed a full PICO, initially applying it within MEDLINE and then translating it into PsycINFO, PsycArticle, Scopus, ProQuest, Ovid Emcare, Google Scholar. However, this produced a very large number of potential sources (270017), most of which were irrelevant to the review purpose and because they could not distinguish appropriately between seclusion and restraint, as used in Western healthcare systems and pasung. For the above reasons we only used “P” within the PICO. The review did not limit the timeframe on publications, as there are very few articles on this topic and we believed, therefore, that there was significant value in including an extensive search timeframe. A grey literature review was also undertaken and will be reported elsewhere in a further publication.

Inclusion criteria were all studies of the following:

1. Pasung-based populations (with or without a firm diagnosis of mental disorder) in the community setting;
2. Peer-reviewed literature published in the English or Indonesian languages, all available up to and including 2019;
3. Empirical studies, systematic reviews (both quantitative, qualitative, and mixed methods studies) or other identified reviews (e.g., narrative, scoping, rapid);
4. Focus on interventions, including those that aimed to: (1) improve diagnosis, investigation, treatment, monitoring and management of pasung; (2) improve pasung management programmes to consumers and carers, health promotion interventions, and interventions designed to improve treatment compliance; (3) improve health care processes, e.g. engagement, follow-up and appointments with mental health services or psychotherapy; and (4) promote consumer and carer involvement.

Exclusion criteria were all studies:

1. Not available in English or Indonesian languages;
2. Non-peer-reviewed literature;
3. Editorials, opinion pieces, letters to the editor, books or book chapters, conference posters, Conference proceedings, formal media and social media;
4. Seclusion in the hospital or other social institutions;
5. Not specific to pasung-based populations.

### Screening process

Titles and abstracts of all results were screened and double-checked based on the agreed inclusion and exclusion criteria. Full-text peer-reviewed papers were further double-screened, with checks of the reference lists of included studies for any further potentially relevant papers, and included if consensus was confirmed by the researchers. At this stage we also activated an alert for further publications until 31 December 2019. Two authors (MTH and SL) independently reviewed the titles and abstracts; then all four authors (MTH, SL, EM, CO) reviewed the full texts against the inclusion/exclusion criteria and performed the quality ratings. For sources that were in Bahasa, the first author undertook each of the screening steps, with collaboration from 1 PhD student and 1 Master of mental health nursing specialist who also spoke fluent Bahasa; each of these individuals performed independent selection and rating. The research team then undertook detailed discussion, making a final determination about the inclusion of these studies. Several of these sources had an English language abstract also available, which aided in understanding the overall intent and steps undertaken for each study. Agreement on final included sources and ratings was reached following robust research team discussion.

### Data extraction and analysis

Data extraction was led by the first author and checked by all research team members. The following data were extracted for each peer-reviewed empirical study: Authors, Year, Country, Study Type, Aims and Methodology, Population(s)/Setting, Number of Participants/Methods of Recruitment, Data collection and Data analysis methods, Main findings, and Limitations. For reports and discussion papers, the extracted information included: Authors, Date, Country, Main purpose, How pasung was defined, Study population demographics and characteristics, Proposed solutions.

## Quality ratings

Only empirical studies underwent quality ratings, using internationally recognised rating scales matched to study type. The CASP (Critical Appraisal Skill Program) [21] was used for qualitative studies. For the before-after studies, we used the relevant JBI (Joanna Briggs Institute) tool [22]; and for the survey, the Critical Appraisal for Surveys developed by Centre for Evidence-Based Management [23] as no instrument was available from the suite of CASP tools to rate these two study types. All quality rating instruments were used to evaluate the methods, results, and value of each study. However, the quality rating process was not intended to exclude low quality studies but to evaluate the quality of the evidence.

Four reviewers (SL, EM, CO and MTH) assessed the English articles (n = 24). All articles were reviewed independently by at least two reviewers, with any differences in rating discussed by the group and consensus reached. CO and MTH reviewed 1 Case Control study, 4 Before-After studies, 1 Cross Sectional study, and 12 qualitative studies. SL and EM reviewed 12 qualitative studies and 5 survey studies. For articles only available in Bahasa (n = 10), quality ratings were undertaken by MTH and two further reviewers who were native for Bahasa. A similar process was undertaken for resolving any differences in ratings. MTH then met with SL to discuss and moderate the ratings. This involved MTH reading out (translating) pertinent sections of the full papers to SL, and SL asking detailed critical questions to clarify the stance taken. Most articles in Bahasa also included an English version of the study abstract, which aided this process.

## Underpinning model

The Socio-Ecological Model (SEM) underpinned this systematic review analysis because of its suitability in understanding this pervasive issue at various social systems levels and exploring any intersections and influences across and between those levels [24, 25]. In addition, no single factor could clearly generate the reasons for family and community to conduct this practice. All factors have potentially had an immense impact on this practice before, during and after pasung. Therefore, using SEM was appropriate as the model incorporates diverse lenses. The SEM was used to examine the four-layer factors adopted by the Centers for Disease Control (CDC) [26]; i.e. individual, relationship, community, and societal factors associated with reasons for pasung and expectations about the management of pasung to fill gaps in unmet needs.

## Results

### Data characteristics

**Fig. 1:**
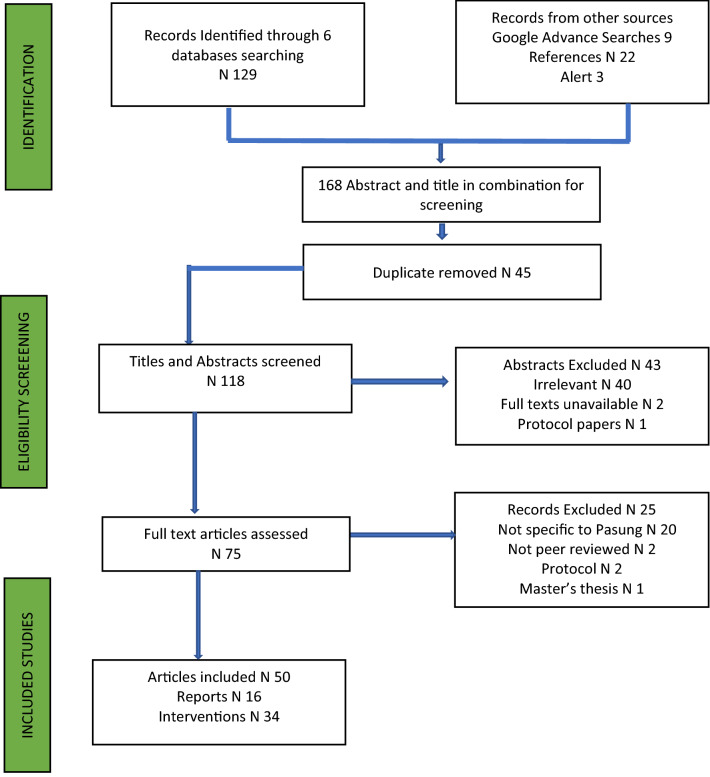
PRISMA representing the number of records retrieved at each stage of the review

The database search retrieved 168 records in combination (Fig. 1). After removing duplicates (n = 45), there were 118 abstract and titles (50 Primary papers, 22 References, 9 Google scholar, 3 Alerts). Review of the titles and abstracts resulted in the exclusion of a further 46 records (irrelevant 40, full text unavailable 2 and protocol paper 1). For the remaining 75 articles, full-text papers were reviewed for eligibility against the inclusion and exclusion criteria. Of these, 25 articles were excluded, with 20 articles not specific to pasung, not peer-reviewed (2 articles) and two protocol articles, and one thesis. Fifty (n = 50) peer-reviewed articles were included in the final review.

Most articles were written by researchers and other stakeholders based in Indonesia (76%) followed by Asian and African countries (Table 1). Of the 50 articles, 16 were reports/discussion papers and 34 were empirical studies of which more than half were qualitative studies, followed by fewer survey studies and those using other quantitative designs; with no randomised control trials (Table 2) (see Additional file 1: Table S1 and Additional file 2: Table S2 for Details of Studies).

**Table 1:** Number of Articles by Country

Country	N	%
Indonesia	34	68
Ghana	2	4
Ethiopia	1	2
Japan	1	2
Singapore	1	2
China	2	4
East Timor	1	2
India	2	4
Philippines	1	2
Somalia	1	2
Chad	1	2
LMIC	2	4
Pakistan	1	2

**Table 2:** Summary of articles by type of methods, intervention type and quality rating

	**N**
Type of methods	
Before–After	4
Case–Control Type	1
Cross-sectional	1
Survey	7
Qualitative	21
Quality rating	
Good (8–10)	11
Fair (3–7)	11
Poor (1–4)	12
Intervention type	
Psychoeducation as a single method and as a combination with the decision care making	2
686 Program (China) unlocking program	1
Community-based program with the culturally sensitive and respectful mental health model	1
Indonesian Free Pasung Program with community mental based approach	1

### Results of the Quality Rating Process

#### Qualitative studies

There were 21 qualitative studies which comprised 14 articles in English and 7 in Bahasa. The CASP qualitative studies quality assessment tool contained 10 questions. Only 1 study was assessed as meeting sufficient quality across all 10 criteria [4]. Question 1 asked ‘Was there a clear statement of the aims of the research?’ and question 2 asked ‘Is a qualitative methodology appropriate?’ Most of studies fulfilled these criteria except for 2 studies where the goal of the study was unclear. Question 3 asked ‘Was the research design appropriate to address the aims of the research?’ Seventeen studies met this criterion, with insufficient detail in 4 studies to confirm whether this criterion was met. Question 4 asked ‘Was the recruitment strategy appropriate to the aims of the research?’ Fifteen studies met this criterion, with insufficient detail about how recruitment occurred and the appropriateness of the sample selection (inclusion and exclusion criteria) in 6 studies. Question 5 asked ‘Was the data collected in a way that addressed the research issue?’ Only 13 studies stated in sufficient detail how the data was collected; for example, by audio recording, focus group discussion or in-depth interview. However, across the 21 studies, few clearly explained the setting or detail about specific questions or interview guides used by the researchers; none explained how saturation was reached. Question 6 asked about the relationship between researcher and participant. Only 2 studies provided specific detail; the relationship was unclear in 16 articles, and 3 studies provided no information about this criterion. Question 7 assessed ethical considerations. Almost half of the studies (n = 9) made no mention or providing insufficient detail about ethical issues. Some studies stated that they gained ethical approval but there was no further explanation about how the researchers protected privacy and confidentiality, or gained consent of participants. This was particularly noted for studies available only in Bahasa. Question 8 asked about the rigor of data analysis. Only 10 studies met this criterion; 9 stated the type of data analysis used (e.g. thematic analysis, Colaizzi’s method) but provided very limited information about the analysis processes followed, and 2 studies provided no information. Most qualitative studies (n = 14) available in English provided an adequate statement of findings (question 9), and some information about the strengths and limitations of the results. However, all studies available only in Bahasa did not mention this and only described theories and ideas that supported their arguments and not in the opposite view. The articles also did not mention how validity and reliability of findings was assured, or triangulation processes. Question 10 asked ‘How valuable is the research?’ Most studies (16 out of 21) were assessed as valuable, with this being unclear in only 5 studies, due largely to insufficient detail in the discussion, and limited or no mention of implications for future research or potential for wider application of the findings.

#### Before-after studies

There were four before-after studies (quasi-experimental) with 9 questions from the relevant JBI assessment tool to rate their quality. Across all articles, few of the criteria were met and many were unclear. For instance, in question 2 (Were the participants included in any comparisons similar?), question 3 (Were the participants included in any comparisons receiving similar treatment/care, other than the exposure or intervention of interest?) and question 7 (Were the outcomes of participants included in any comparisons measured in the same way?), these criteria were unclear for all studies. Most studies did not have a control group (question 4). Other weaknesses of these studies were the lack of complete follow-up, lack of detail about follow-up when it was reported, and limited or no information about strategies to deal with loss to follow-up. The appropriateness of the statistical approach was unclear for 3 out of 4 studies. This result then impacted the outcome reliability, with only 2 of the 4 studies meeting this criterion.

#### Survey studies

There were 7 studies which used a survey design, consisting of 2 in Bahasa and 5 in English. The CASP quality rating scale for surveys comprised 12 questions: however, question 12 (Can the results be applied to your setting/organization?) was not rated due to not being relevant for this review. No studies met all the criteria; only 3 of the 7 studies met most criteria; and the remaining 4 studies met between 2 and 4 criteria. Only 2 studies provided confidence intervals.

#### Cross sectional studies

The JBI scale for assessing cross-sectional studies consisted of 8 questions. Only one study was included, and only 3 of the 8 criteria were met. Inclusion criteria were not clearly defined, subject setting was not clearly described, and the researchers failed to describe confounding factors and strategies to deal with these. However, it did mention how the researchers aimed to maintain validity and reliability, using selection criteria for the participants, and use of a valid outcome measure.

#### Case control studies

The CASP assessment tool was used to rate the one included case control study which met 9 of the 11 criteria. Size of treatment effect and management of potential confounding variable were unclear. (see Additional file 3 for Quality Ratings Tables).

### Key findings

Key findings are presented in Table 3. These informed the narrative synthesis that follows.

**Table 3:** Key findings

No.	Author and year	Key findings
1	Alem (2000) [27]	Care providing in Ethiopia does not seem to be in accordance with UN Declaration of Human Rights. As per most low-middle income countries that are unable to fulfill the basic needs of their citizens, it appears that the mental health system in Ethiopia will not change in the foreseeable future
2	Anto and Colucci (2015) [28]	The story of Anto who was shackled several times since he was a young age. Anto achieved good grades whilst in school. However, he was very shy and lacked confidence and was bullied during his school years (fancy boy); this led him to become a person with low self-confidence. As a teenager he suffered from depression which became worse over time. The symptoms re-appeared when he was working at the paper factory and also while he was sitting at the university. The family decided to place him in pasung as they were afraid Anto would hurt himself or disturb the neighbors. After 3 times in pasung he was finally freed by the Free Pasung Program from the local psychiatric hospital and shared his story with others, using various forms of art to express his experiences, to help them to be free from pasung
3	Asher et al. (2017) [7]	Most of the participants with schizophrenia and their caregivers had personal experience of the practice of restraint (pasung). The main explanations given for restraint were to protect the individual or the community, and to facilitate transportation to health facilities. These reasons were underpinned by a lack of care options, and the consequent heavy family burden and a sense of powerlessness amongst caregivers. Whilst there was pervasive stigma towards people with schizophrenia, lack of awareness about mental illness was not a primary reason for restraint. All types of participants cited increasing access to treatment as the most effective way to reduce the incidence of restraint
4	Broch (2001) [6]	Stigma was central to how people in the village reacted to the mentally ill person (gila betul). Most of villagers believed that the person was possessed and dangerous, that mental illness was an evil spirit (Jin) that should be cured by spiritual or traditional healers. Some of them also thought that mental illness was a disease
5	Buanasari et al. (2018) [29]	Pasung use with parents with mental illness had a clear psychosocial impact on adolescents. Role changes occurred when the teenager became a breadwinner and caregiver of the parent. The experiences felt by adolescents were the changes in every aspect of their life in the form of roles, psychological and social conditions. Three themes: 1. Changed life due to having a mentally ill patient with pasung 2. Reciprocity as the reason of taking care of the parent 3. Positive meaning of life with having mentally ill parent with pasung
6	Daulima et al. (2019) [30]	The family breadwinner felt a lack of confidence due to role change after having their family member in pasung. In addition, the family had more problems with family finances as they now had to take care of the person. This meant someone needed to look after the person all the time and could not go to work. Another reason why the person became a burden was the high cost of medication and treatment. However, the neighbors and the environment were fully supportive for the breadwinner to continue working by giving support for casual work like domestic chores. This support increased their confidence and was a strong basis for them to continue their role as head of the family
7	Daulima (2018) [31]	The validity and reliability results showed that the content of this instrument is valid once improvements had been made to the statement item numbers 16 and 17. It was also shown to be reliable by the consistency of the responses with an alpha value of .729. That is, responses to the instrument are consistent and are reliable measures of the level of intention of the mentally ill patient’s family to use pasung
8	Dewi et al. (2019) [32]	The study demonstrated that family burden was significantly lower among those who received the combination of the two therapies (family psychoeducation and care decisions without pasung) compared to only family psychoeducation (p < .05). Those therapies decreased the family burden into the low category
9	Eka and Daulima (2019) [33]	Ten studies were found. There were 3 main factors related to pasung: 1. Factors that originated from the person such as aggressive, wandering, homicide. These behaviors were triggered by the medication drop out due to financial problems and health service inaccessibility. Proposed solutions were educating the person about medication compliance 2. Factors that originated from the family including financial burnout, emotional instability, helplessness, lack of knowledge, dissatisfaction with health services, and the fear that the person would do harm to others or self-harm. The family decided to use pasung as a treatment after a family discussion, and pressure from the community; hence, perceived community stigma was a prominent factor in their decision-making. Proposed solutions were family education about medication compliance to overcome stigma about its use 3. Factors that originated from the community including stigma and discrimination, which caused increased family burden. This meant families often decided to use pasung instead of mental health services. The community also commonly played the main role in deciding pasung. Proposed solutions were community empowerment; in particular, empowerment of community leaders with influence in decision-making (though a limited description of the empowerment process was provided)
10	Firdaus (2016) [34]	1. There are local regulations in Jogjakarta, Indonesia to protect people with schizophrenia and to reduce the practice of pasung in the form of the gubernatorial regulation number 81/2014 aim to improve mental health knowledge 2. Community-based services have been used in several mental health services with using the community volunteer to identify people with schizophrenia in its territory 3. There are some obstacle in fulfilling the right for a mentally ill person as mental health is not the main priority proved by a small budget and cases where many mentally ill wanderings on the street and neglected at a nursing home
11	Guan et al. (2015) [2]	96% of patients were diagnosed with schizophrenia. Prior to unlocking, their total time in pasung ranged from two weeks to 28 years, with 32% having been locked multiple times. The number of persons regularly taking medicines increased from one person at the time of unlocking to 74% in 2009 and 76% in 2012. Pre-post tests showed sustained improvement in patient social functioning and significant reductions in family burden. Over 92% of patients remained free of restraints in 2012
12	Hall (2019) [35]	People with mental illness in Timor-Leste were found to face widespread, multi-faceted sociocultural, economic and political exclusion. They were stigmatized as a consequence of beliefs that they were dangerous and lacked capacity, and experienced instances of bullying, physical and sexual violence, and confinement. Several barriers to formal employment, educational, social protection and legal systems were identified. Experiences of social inclusion for people with mental illness were also described at family and community levels. People with mental illness were included through family and community structures that promoted unity and acceptance. They also had opportunities to participate in activities surrounding family life and livelihoods that contributed to intergenerational well-being. Some people with mental illness benefited from disability-inclusive programming and policies, including the disability pension, training programs and peer support
13	Hartini (2018) [36]	The result shows that better knowledge about mental health was associated with lower public stigma toward people with mental disorder. Significant differences in stigma toward people with mental illness were also found across groups according to age, sex, experience of contact, history of mental disorder, attitude toward pasung, marital status, and income level. Age was negatively correlated with stigma; people were more tolerant as they got older. Married individuals were more tolerant. A history of mental illness in the family equated to greater tolerance. No marked differences in level of stigma were found across groups according to educational level
14	Helena (2018) [37]	Pasung has a physical and psychosocial impact on people with mental disorders in adapting to society. Four themes: (a) Withdrawal from others as an initial manifestation of release from pasung; (b) Biopsychosocial changes after pasung that act as an impediment to performing a social function; (c) Improved social function through the optimization of support systems; and (d) Satisfaction with life as a result of social adaptation
15	Idaiani and Raflizar (2015) [38]	The most profound factor contributed to pasung practice was low social-economic status. The low socio-economic families have 2–3 times greater risk than the middle and high oncome families. In contrast, a geographic area with inaccessibility to health facilities and high mental health literacy have no significant relationship with the pasung
16	Irmansyah et al. (2009) [8]	The focus of the Indonesian Constitution on rights pre-dated the Universal Declaration. Indonesia has ratified relevant international covenants and domestic law provides an adequate legal framework for human rights protections. However, human rights abuses persist, are widespread, and go essentially unremarked and unchallenged. The National Human Rights Commission has only recently become engaged in the issue of protection of the rights of persons with mental illness
17	Jones (2009) [39]	Services created by non-governmental organizations in these contexts are a drop in the ocean compared with what is needed. In all areas mentioned, most people with severe mental disorders remain unrecognized, untreated, and unable to access services. Non-government agencies cannot be a substitute for effective government strategy and action. But they might sometimes be a stimulus; for example, emergency mental health services developed by the International Medical Corps and other national and international agencies have sometimes become seeds for effective longer-term models of care in a number of countries
18	Katuuk (2019) [9]	Three themes were expressed from the family who used pasung for their family member: 1. The helpless feeling of family in adapting to the mental state of a person in pasung, as the family were unable to provide continuous medication. The family must share the funding for the treatment and other family members’ need, like school and food; 2. Ensuring security was the main reason to justify returning the person to pasung and to cover up the guilty feelings for doing so; and 3. As a substitution for re-pasung and feeling guilty, family fulfilled the person’s the basic needs and reduced the length of time of pasung, such as releasing the person temporarily, but with close supervision by the family
19	Laila (2018) [40]	Family members and society in general perceived that pasung is necessary for security reasons due to the person’s aggressive behavior (e.g. physical violence towards family members, damaging neighbors’ property and stealing food). Family often did not respond to the patient’s request to be released from pasung. They felt insecure and helpless when the person was not in pasung and wandered outside the house. Family members had financial constraints that stopped them from seeking mental health care, and they were also dissatisfied with the available services. Health care workers underlined the poor knowledge and misconceptions of schizophrenia in the community
20	Laila (2019) [41]	The person’s aggressive or violent behavior (AdjOR: 4.49, 95%CI: 2.52–8.0), unemployment (AdjOR: 2.74, 95%CI: 1.09–6.9) and informal employment (AdjOR: 2.5, 95%CI: 1. 1–5.84) in the family, and negative attitude of the family towards the person (AdjOR: 2.52, 95%CI: 1.43–4.43) were associated with pasung. The person’s aggressive or violent behavior (PAR = 44.3%) and unemployment in the family (PAR = 49.3%) were the predominant factors for the use of pasung by the family
21	Maramis (2011) [18]	In most countries in Southeast Asia, mental health spending is no more than 2% of the health budget, with 80–90% going to mental hospitals. There are massive workforce deficiencies; few consumer, carer, or other civil-society organizations with a focus on mental health advocacy; inadequate protection of the rights of people with mental illness; few efforts to promote mental health; little in the way of rehabilitation services or efforts to promote social and economic inclusion; and treatment services are concentrated in urban areas and often of poor quality, inaccessible, and unaffordable
22	Marthoenis (2016) [42]	Mental health services in Aceh have been improved compared to their condition before the Tsunami, with development programs focused on procurement of policy, improvement of human resources, and enhancing service delivery. The case of Aceh is a unique example where conflict and disaster, and the need for security, serve as the catalysts toward the development of a mental healthcare system. Despite these improvements, some issues such as stigma, access to care and political fluctuations remain challenging
23	Miller (2012) [43]	In Aceh, Provincial health authorities are creating a community mental health program that shifts much of the work traditionally done by psychiatrists to general practitioners, nurses and village volunteers. In rural areas, where resources are limited, training and delivery of care by less specialized health workers shows promise as an effective way to manage demand for support by rural communities where people with mental health conditions are under-served and at greater risk of experiencing pasung
24	Minas and Diatri (2008) [3]	Fifteen cases of pasung, approximately even numbers of males and females and almost all (n = 13) with a diagnosis of schizophrenia were identified; 9 had previously received psychiatric treatment. Duration of restraint ranged from two to 21 years. Travel was the major cost of treatment component cited as unaffordable (nearest available treatment was 6 h away by boat and then road). The most common form of pasung was in a small room or hut. Reasons given for pasung: violence, coming to harm by running away or wandering off, concern about suicide, and unavailability of a caregiver. Affordable and equitable access to basic mental health services seen as the only effective and sustainable solution
25	Molodynski (2017) [20]	Coercion remains a dominant theme in mental healthcare and a source of major concern in many countries. While the presence of coercion is ubiquitous internationally, it varies significantly in nature and degree in different countries and is influenced by a variety of factors. Recent reports have raised concerns about physical restraint and the increasing use of legislation in high income countries. At the same time, a recent Human Rights Watch report on pasung (the practice of tying or restricting movement more generally) in Indonesia has served to highlight the plight of many in middle- and lower-income countries who are subject to degrading and dehumanizing ‘treatment’
26	Ndetei and Mbwayo (2010) [14]	Lack of knowledge of the cause of mental illness or the fact that such conditions can be treated may lead to mistreatment of patients with mental illness. It is possible that chaining is practiced more widely, and in more countries, than is realized. There is therefore a need for an audit to determine just how common this practice is—a practice which has no place in contemporary African psychiatry
27	Nurjannah (2015) [65]	‘Connecting care’ as the core category to describe a model of care that involves health professionals and non-health professionals, such as family members. Four main factors influence care-providers’ decision-making: competence, willingness, available resources and compliance with institutional policy. Health professionals are influenced most strongly by institutional policy when deciding whether to accept or shift responsibility to provide care. Non-health professionals base their decisions largely on personal circumstances. Jointly made decisions (between the various stakeholders) can be matched or unmatched. Unmatched decisions can result in forced provision of care, increasing risks of human rights violations
28	Patel and Bhui (2018) [44]	A rights-based approach must enforce well-established international human rights conventions, and scale-up comprehensive community services around the needs and preferences of people affected by mental disorders
29	Patel et al. (2009) [45]	The plan proposed is based on the socio-cultural, epidemiological and health system contexts of a specific location in one country. Although ‘one size does not fit all’ in health-system interventions, such a plan may serve as a blueprint for other contexts, following appropriate modification and adaptation to ensure its feasibility, acceptability and relevance
30	Patel Saxena (2018) [46]	Three measures are proposed: first, balancing the focus on treatment, rehabilitation, care, and recovery with an equal emphasis on the promotion of mental health and the prevention of mental disorder, particularly interventions early in the life course; second, adopting a staging approach to the identification and diagnosis of mental disorder, recognizing the potential benefits of intervention at each stage; and third, embracing diverse global experiences of mental health and disorder, to tailor the range of inter-ventions more appropriately and promote mutual learning. Key terms for defining the scope of mental health are also proposed
31	Puteh (2011) [47]	Fifty-nine former pasung patients were examined. The majority (88.1%) of the patients were male, aged 18 to 68 years. The duration of pasung varied from a few days to 20 years, with a mean duration of 4.0 years. The reasons for applying pasung are many, with concerns about dangerousness being most common. The great majority (89.8%) had a diagnosis of schizophrenia
32	Rahman (2016) [48]	The nurses had been carrying out their role as executors of nursing care policy, as the direct nursing as caregivers, and were providing direct nursing care to people who had experienced pasung and their families, as well as continuing therapy for ex-pasung sufferers, and as educators, collaborator and also educating the family The nurses faced a difficult challenge in implementing free pasung program, including: 1. Family and community rejection 2. An emotional expression such as grieving, frustration, give up 3. The absence of a caregiver 4. Illiteracy on mental health 5. Unavailability of an anti-psychotic drug 6. No partnership and multisectoral coordination 7. Multiple tasks
33	Rasmawati (2018) [49]	The pasung patient potentially lost the support from families (in particular their spouse) due to being unable to fulfil their basic needs, their aggressive behavior, and being judged as unable to recover like a ‘normal’ person. Divorce has an additional impact on the people in pasung. Grieving is the first response to separation from children and spouse. Most of the respondents were left by their spouse due to their mental health problem. The problem became worse when the person could not find a new partner due to their mental illness and financial barriers
34	Read (2009) [4]	Chaining and beating of the mentally ill was found to be commonplace in homes and treatment centers in the communities studied, as well as with-holding of food (‘fasting’). However, responses to mental illness were embedded within spiritual and moral perspectives and such treatment provoked little sanction at the local level. Families struggled to provide care for severely mentally ill relatives with very little support from formal health services. Psychiatric services were difficult to access, particularly in rural communities, and also seen to have limitations in their effectiveness. Traditional and faith healers remained highly popular despite the routine maltreatment of the mentally ill in their facilities. Caution is suggested when taking a moral perspective on rights and responsibilities in the context of pasung use in this context, as this may be used to justify the maltreatment of people with mental illness, as this research has suggested
35	Reknoningsih (2014) [50]	Most caregivers were poorly educated (primary level and not educated). There were five themes: 1. The family felt physically exhausted and emotional distress caring the pasung person. 2. The family’s emotional burdens and being physically exhausted were reasons given for re-restraining their family member. 3. Further family difficulties arose due to the burden of pasung management, and the person’s aggressive behaviour. As a result, re-pasung was the main option for the family. 4. The families have internal and external support for caring for the person - Either material support like money and staple food or external support like free insurance and free medication. 5. However, families get more spiritual understanding as part of caring for the person whilst in pasung in the form of experience of spirituality, being closer to God and recognition
36	Riany (2016) [51]	The interviews revealed five related themes about autism: 1. Understanding about autism; 2. Causes of autism (traditional cultural beliefs about pregnancy, belief in karma and God’s plan); 3. Beliefs about how best to care for a child with autism (traditional and medical treatments, education, good parenting; 4. Reactions to having a child with autism (self-blame, shame, expectations of stigma); 5. Parenting a child with autism (impact of shame, parenting practices and use of coercion. Overall, despite many understanding the underlying medical causes of autism, their traditional cultural beliefs led many to stigmatise children with autism and their family, creating increased isolation in the community
37	Sa’ad and Bokharey (2001) [52]	A total of 100 patients in pasung at shrines were treated, with the age range from under 9 to those above 70 years, with most aged between 10-29 years. Most of the patients had a mental health condition such as schizophrenia, depression and epilepsy
38	Saribu and Napitulu (2009) [13]	The Indonesian legal system/national laws which regulate the right of persons with the mental disorder include: 1. Law No. 23 of 1992 concerning health 2. Law No. 39 of 1999 concerning human rights 3. Law No. 4 of 1997 concerning a person with disabilities 4. Pela code of Indonesia (is pasung could be categorized as a delict (violation of law)?) 5. Indonesian criminal procedure code (pasung could be classified as a criminal deed; however, up until now, no perpetrators of pasung have been punished by the courts
39	Stratford (2014) [53]	Ministry of Social Affairs (MoSA) has been able to utilize the extensive experience and skills of its Australian partners to enhance implementation of the plan. The success of the collaboration of Mind and Australia Asia Mental Health (AAMH) program with MoSA has been achieved through a rigorous concern to ensure that concepts of psychosocial rehabilitation and approaches such as the recovery approach which originated in developed western nations are relevant and applicable to the Indonesian context
40	Suharto (2014) [54]	1. The age of people in pasung is between 13 and 70 years, dominated by the male (3:1), with the length of illness from 2 to 35 years, the incidence of relapse from 1 to 7 times during the illness, and the average duration of pasung is 8.5 months. 2. The majority of families resorted to pasung as the preferred treatment due to the high cost for the medications. This was as direct costs to buy medications and pay the mental health staff and indirect costs for transportation (most mental health services are located in the central city which sometimes took a day’s journey to go back and forth). 3. Most caregivers were parents (mostly their mother), age 50 or more, low educated (primary level and uneducated), working as farmer/gardener/Warong. There is a significant relationship between education level and age of family with the social function of the family. 4. Pasung practice is not solely negligence of family to give care to their family member but also the failure of the government to provide mental health services at the primary level
41	Suhron (2017) [55]	The mean score before the family psychoeducation intervention was 21.6; and after family psychoeducation, the mean increased to 29.1. The Wilcoxon test showed ρ value = 0,000 < α = .05 which meant there were differences in the ability of families to care for people with mental disorders before and after family psychoeducation
42	Suhron et al. (2018) [56]	The caregivers were mostly female, average age 27, a third of caregivers were not working, more than three quarters (83%) gained primary education or lower, more than three quarters (80%) lived in a remote area and nearly a half of caregivers were parents. Cultural values effect the family’s role which indirectly affects the ability of the family in caring for the person
43	Suryani et al. (2011) [5]	The development of a community-based, culturally sensitive and respectful mental health model can contribute to positive mental health outcomes. The traditional medical, hospital-based, psychiatric model currently practiced in Indonesia, and arguably in other countries, possesses an inherent inability to provide a holistic and equitable service to this population and in this cultural context. after 19 months of holistic treatment, none of the patients were confined in pasung, and only 2 required further intensive treatment. Community education forums and workshops to educate them about mental health issues in a meaningful and respectful language that was a aligned with their culture and customs was effective (500 per month attendance). Mutual support groups for families and community members were also established
44	Tanaka (2018) [57]	The findings highlight the culturally and socio-economically specific contexts, consequences, and impact modifiers of experiences of stigma. Participants emphasized that PMHP face stigma because of the cultural traits such as the perception of mental health problem as a disease of the family and the tendency to be overly optimistic about the severity of the mental health problem and its impact on their life. Further, stigma was experienced under conditions where mental health care was not readily available and people in the local community could not resolve the PMHP’s mental health crisis. Stigma experiences reduced social networks and opportunities for PMHP, threatened the economic survival of their entire family, and exacerbated their mental health problems. An individual’s reaction to negative experiences can be fatalistic in nature (e.g. believing in it is God’s will). This fatalism can help PMHP to remain hopeful. In addition, traditional communal unity alleviated some of the social exclusion associated with stigma
45	Tay et al. (2017) [58]	The first case report examining the prolonged use of pasung in a developed urban setting. Illness factors, family dynamics, stigma, lack of mental health literacy and cultural roles contributed to her chaining. Despite Singapore’s excellent infrastructure, highly educated public, accessible professional psychiatric treatment and overall modernism, there remains a minority of psychiatric patients who are beyond the reach of the treating team
46	Ulya (2019) [59]	Pasung and mental disorder produce a vicious circle which is difficult to break, particularly when the perpetrator is the family. On the one hand, family is the key to care for the person but on the other hand the family have limited resources and sometimes are exhausted by their caring responsibilities. Pasung is more common in rural locations and among lower socio-economic groupings. Stigma towards mental illness is prominent. When adopting an ethical viewpoint, pasung must not be used as a substitution for treatment as it violates human dignity and human rights. The basic moral principles in bioethics show that ill-treatment and coercion fail to adhere to four basic rules (i.e. respect for autonomy, beneficence, non-maleficence, and justice). There is no justification for the view that unregulated coercion is a form of treatment for mental disorders. Any attempt to justify such coercion violates principles of international and national health law. More integrated community programs are needed to address stigma and support families
47	Vijayalakshmi et al. (2012) [60]	Gender differences were clearly evident. Although, subjects enjoyed similar satisfactory levels of fulfillment in the physical dimension of human rights needs, which included food, housing, and clothing, men expressed lower satisfaction than women with perceived human rights needs fulfillment in the emotional dimension. This included fear of family members (# 2 = 9.419,p G .024) and being called derogatory names (# = 8.661, p G .034). Women expressed lower satisfaction than men with perceived human rights needs fulfillment in social and ethical dimensions. This included freedom to leave the home (# 2 = 11.277,p G .010), and sexual abuse by family members (# 2 = 9.491,p G .019). Men felt more discriminated against than women due to perceptions of mental illness in the community domain (# 2 = 10.197,p G .037)
48	Wirya (2017) [12]	Four areas of thinking are proposed: 1. The discourse of madness in this article is defined as a set of statements that has institutional strength, which means a set of statements that have a profound influence on the way we act and think individually. The act is to do pasung 2. Pasung is a part of madness discourse formed by the power, knowledge and social structure of community resulting pasung as a rational act to control someone who they called as crazy 3. This research showed that the discourse of madness that promotes the truth regime of rationalism and produces pasung as a part to control of the those deemed as ‘crazy’ is a crime that must be dealt with through a replacement discourse. Human agents can build a replacement discourse which uses chaos theory and existentialist psychological thought 4. Even though psychiatric therapy tried to eliminate and replace this current discourse, psychiatry itself assumes the insane as an object that needs to be controlled
49	Wulandari et al. (2019) [62]	Several themes were identified, for example: 1. The reluctance to be re-pasung. The patient refuses to be subjected to pasung and stating that pasung was a horrible experience 2. The demand to have interaction with other people. The patient wished to interact freely and making friends 3. The sense of being ignored due to stigma. The patient felt being exiled, sad and trauma because of stigma
50	Yusuf and Tristiana (2018) [10]	1. Most of the caregivers were parents, followed by spouse and other relatives. 8 from 9 respondents were poorly educated (primary level) and work as a casual worker (farmer, gardener) with some were unemployed 2. Most patients had been subjected to pasung from 1 to 24 years. 3. The intention of the family using pasung was to control patient aggressive behaviour (to others, themselves and neighbourhood), for curing, avoid wandering and because they could not continue to care for the patient. The family also had no other options as they had to work to feed their family and the person in pasung. In addition, they had limited financial resources, and endured high cost of treatment 4. The decision to use pasung came from families, neighbours and the communities. The communities and the family work together to build the shelter for pasung like creating the hut, making a window from iron 5. The impacts of pasung were atrophy, contracture, and psychological aspects (depressed, isolated, trauma, hate)

### Narrative synthesis using the socio-ecological model

#### Individual-level

Sixteen articles discussed individual-level factors as a risk and or primary cause for pasung practice. This individual level included biological and personal history factors identified in the literature that increased the risk of the person being subjected to pasung, i.e. age, marital status, sex, employment, education, mental health history, medication history, and aggressive behaviour. The literature reporting rates of pasung generally identified that people subjected to pasung were predominantly male, age 18-60 years old, with a previous mental illness, unemployed, with a past pasung history, and a low-level of education. Although two articles reported similar rates of pasung for males and females, overall, there was no correlation between sex and pasung practice. However, in most articles, males were more likely to be subjected to pasung. Approximately 3 out of 4 people in pasung were male [41, 47, 54]. Younger men with schizophrenia were at greater risk of being subjected to pasung than young women [3, 41, 47, 52]. Although the association remains unclear, this use of pasung in young men appears to be related to more aggressive behaviour or violence developed at a younger age.

In most articles, a person in pasung was more likely to be single with almost 30% being divorced. However, it is not possible to state a direct correlation with having a mental illness and marriage breakdown from this data [41]. Nevertheless, one study [49] described that many people who experienced pasung had been left by their spouse or partner because of having a mental disorder and experiencing personal hardship.

Most people in pasung had a mental disorder history, with 89–96% diagnosed with schizophrenia [2, 47] and the duration of illness ranging from 1 to 35 years. Despite studies finding that pasung was predominantly experienced in adult populations, some studies also found that pasung occurred in school-age children (those around 13 years old) and some were older than 60 years of age [47, 54].

For the duration of pasung, defining how long the person was in pasung was problematic because it was unclear when pasung initially began and whether the person was released for short periods and then placed back in pasung, as explained by Asher et al. [7]. Reports of duration varied across studies, with Laila et al. [41] stating that individuals had been subjected to pasung from 1 day to 12 years (mean 1.3 years). Another study showed a longer duration of pasung; up to 24 years [10]. The history of pasung use is also important to highlight as some articles reported that more than 30% of individuals subjected to pasung had past experience of being restrained one or more times [2, 7, 9, 47].

Most pasung use with people with a history of mental illness was believed to be because they had failed to continue their medication [3]. Medication nonadherence was suggested to be the cause of illness relapse in 96.8% of patients and exhibition of aggressive behaviour [41]. Treatment drop-out was also reported by Minas and Diatri [3] who found that 9 out of 15 people in pasung had previous treatment with mental health services. Medication non-adherence was also seen to be related to the inability of the family to provide regular medication to their family member due to factors including cost and the lack of availability of family members to assist PWMD to go to mental health services [9]. Another factor identified as a root cause of medication discontinuation was refusal by the PWMD, with Asher et al. [7] reporting the PWMD was usually restrained (tied up) in the journey to the health facilities as they often refused to be medicated.

Laila et al. [41] reported that PWMD with aggressive or violent behaviour were five times more likely to be subjected to pasung than non-aggressive PWMD. The study by Reknoningsih, Daulima and Putri [50] echoed the above finding and restated that aggressive behaviour led the family to use pasung on more than one occasion with relatives. Other studies confirmed that violence was a reason for the family to use pasung [3, 47].

In addition to individual level factors relating to the demographic and behavioural characteristics associated with pasung, we also identified literature reporting the effect of pasung on individuals. As a result of the prolonged duration of pasung, some people were severely negatively impacted, both physically and mentally. A few studies mentioned the impact of pasung [7, 10, 29, 37, 49], but no studies precisely measured the physical and psychological impact of pasung for the PWMD, their family or the community, for instance, in the long or short term. Asher et al. [7] reported that two PWMD had physical injuries resulting from restraint, including bruising, contracture, and deformity of the leg. Mostly, the injuries happened because the person in pasung could not move freely or because they were held in wooden stocks in one position for a long time.

Similarly, another study by Yusuf and Tristiana [10] found that many PWMD subjected to pasung in Indonesia experienced atrophy and contracture, mostly in the lower limbs. Another finding was that many PMWD experienced cachexia (wasting syndrome) due to being under-nourished or denied food and water. One study also reported that the person in chains was often tied without access to food [4]. Pasung impacts were not only physical; studies reported that they also involved the person in pasung experiencing psychological problems. Yusuf and Tristiana [10] noted that the person experienced depression, isolation, and hatred towards those who put them in pasung. Many respondents in their qualitative study also reported being traumatised by the pasung experience and avoided contacting other people as a consequence of that experience. In another paper, community leaders and community health workers also highlighted the detrimental psychological impacts of being restrained and acknowledged that it could make the illness worse or increase the risk of violence towards others, including those the PWMD knew very well [7].

#### Interpersonal relationships

Interpersonal relationships consisted of the relationships between the person experiencing pasung and their community, spouse/partner, and family, and in particular the interconnection of a person’s behaviour and perceived risk from family. At the socioeconomic level, adaptation and family burden were also included in this domain.

Reported reasons for using pasung were similar for studies conducted within Asia and Africa where PWMD were tied up for extended periods of time in order to give them medication, transport them to health facilities, or where the caregiver was no longer available [7, 10, 54]. The family were also the most common perpetrators of pasung towards their family members who were mentally ill. This finding was highlighted by two good quality articles. The first article, a quantitative study by Laila et al. [41], stressed that family were the dominant actors in implementing pasung, with mothers being most likely (32.46%), followed by fathers (27.19%), or elder siblings (9.65%). A second study by Asher et al. [7] also found that family members were the main people responsible for instigating pasung. The two articles were conducted in low to middle-income countries (LMIC): Africa (Ethiopia) and Asia (Indonesia). However, findings from these studies were not likely to be representative of the complete picture for the use of pasung in LMIC because both studies were conducted with small samples and in local research sites.

Carers of PWMD in pasung were reportedly poorly educated [50]. Reknoningsih, Daulima and Putri [50] furthermore highlighted that family were predominantly female, usually the mother, aged above 50, and some were unemployed or were a daily hire worker such as a gardener or farmer. Similarly, three further articles described the carer as uneducated or as having a low level of education [10, 54, 56] and living in a rural area. Despite the relationship between carer education and the practice of pasung being unclear overall, one study suggested that level of education is strongly correlated with the social support for the person in pasung [54]. As an example, the family were also reported to frequently reject requests from the person in pasung to release them. This was done out of fear that the person would wander and create further burden for the family. As a replacement for pasung, the family sometimes released the person and used close monitoring and observation to prevent wandering [9, 40].

Perceived risk and safety from aggression was another critical finding frequently identified as an interpersonal factor increasing the use of pasung [2–5, 29, 35, 41]. These articles identified the use of pasung was to protect the family and community and to protect the PWMD themselves. Asher et al. [7], for example, described pasung as the choice taken by the family to protect the individual and to make it easier to facilitate transportation to health facilities. This was supported by Hall et al. [35] who also found that the PWMD often experienced bullying, physical and sexual violence by surrounding communities when the person was wandering. Broch [6] agreed with this finding, stating that PWMD were often ridiculed by the community (mostly children) who called out the words ‘gila betul’ (‘crazy’ in Indonesian). These experiences triggered PWMD to act aggressively, forcing caregivers to restrain the person at home to avoid further problems for the family, community, and the person themselves.

A further interpersonal reason for the use of pasung identified in the literature was to address the burden of caring for their family member in pasung. As reported in nine articles, family members experienced stress and felt powerless as a consequence of caring for the person in pasung, feeling they had to manage the burden alone [4, 7, 9, 10, 29, 30, 40, 50, 59]. For example, Read, Adiibokah and Nyame [4] asserted that families were pictured mainly as being powerless, with extremely limited options and sometimes driven to act out of fear. Similar findings were reported by Yusuf and Tristiana [10], who stated that the decision to use pasung came after a long discussion with and pressure from the community. However, the primary responsible person for making the decision was the main caregiver as a practical response to a challenging situation.

Due to family members’ need to get income, pasung was also sometimes used to control the PWMD to allow the caregiver to attend work. Helena, Daulima and Wardani [37] also identified that the family experienced a psychosocial change after their family member was placed in pasung, mainly if the person had previously been the head of the household or primary breadwinner. In these circumstances, other family members now had to find new ways of gaining income for the family or taking on the role as head of household [29, 31]. Consequently, many families with a PWMD lived in poverty. Low socioeconomic status for families was identified as a risk factor for the use of pasung. Idaiani and Raflizar [38] claimed that families from low socioeconomic backgrounds had a 2–3 times greater risk of using pasung on their PWMD family member.

The family role also had to change, not only because the person was unemployed, but also because of the person’s lack of confidence due to their diagnosis of mental illness [37]. The person’s condition was reported to worsen due to increased feelings of guilt about not being able to fulfil their commitments and role within the family with consequent loss of dignity [4]. As a result, many PWMD lived alone or were left by their partner [49].

Lack of accessible and affordable treatment options appear to underlie many of the caregivers’ narratives. While stigma is another factor that will be discussed separately, its existence was indicated by the assumption that PWMD are likely to be violent or aggressive, and thus have to be in pasung. Another finding from four articles [10, 29, 40, 41] was that pasung was described as a last resort after the family believed that the medication given by mental health services was not helping the PWMD, given the experience of relapse and prolonged illness despite treatment.

#### Community

Communities play an essential role in supporting the recovery of PWMD and also in the overall impact of illness due to stigmatizing, inaccurate beliefs about mental illness. Community factors determined from this review include socioeconomic status (both the person and family, and the community surrounding them if reported), geographical area, cultural identity, social inclusion and integration, literacy, and infrastructure.

Several studies reported that myths and beliefs in the general population about mental disorders are associated with the increased use of pasung [6, 7, 33, 35, 36, 42, 49, 51, 57, 58]. PWMD are often considered dangerous and/or possessed by demons. This includes, for example, the belief that mental illness is related to the person having supernatural abilities, or results from disturbed spirits or black magic. These stereotypes increase discrimination and ostracization by those around PWMD [4, 6, 7, 58]. Other studies reported that some family also believed that the existence of mental illness for the PWMD was god’s will [51, 57]. They viewed having a family member with a severe mental disorder as their destiny because of their past sins or as a temptation. Some family members blamed themselves for having someone in their family with mental illness [51, 62].

Instead of getting proper medical treatment, PWMD in some communities were isolated from social life because they were considered as a ‘family disgrace’ and dangerous. As a result, communities and or families often sought the help of spiritual healers and traditional healers as the first choice for treatment [7, 57–59]. These three articles reported that all families of those in pasung had used traditional medicine, either by a dukun (shaman) or spiritual healer. Unlike the information provided by health workers who treated mental illness as a disease, traditional practitioners often provided misinformation to the family about the causes of illness.

Misleading information was exacerbated by a low level of mental health literacy, which is reportedly associated with pasung, although the nature of the relationship remains unclear [7, 38, 58]. Mental health literacy is linked with stigma, which has been identified as one of the predictive factors of pasung. A study by Hartini et al. [36] showed that better mental health literacy was associated with lower negative stigma toward PWMD. In addition, better knowledge of mental illness was thought to prevent communities from mistreating PWMD [10].

Inaccessibility of health care, particularly mental health care, is a further community level risk factor. Some authors identified that health care inaccessibility is the main reason why PWMD are restrained in Africa [4, 7]. Similarly, in Asia, mental health care access was a key issue around the practice of pasung. Lack of access as the core driver of pasung practice has been reported in three articles [2, 3, 5]. Although all articles were of poor quality, they were consistent in identifying that pasung happens in communities where formal mental health care is inaccessible, and there is a lack of antipsychotic medication.

While some authors identify a lack of access to mental health care as a significant factor, others found this not to be the case. For example, Suharto [54] found that the majority of families resorted to pasung due to the high cost of medications. Costs included direct costs to buy drugs and pay mental health staff, and indirect costs for transportation (most mental health services are located in metropolitan areas and travel from rural areas these metropolitan services could take many hours). Although this article is of low quality, it presents multiple perspectives, including that of families. Tay et al. [58] reported similar access to care issues in an urban setting in Singapore where mental health access is available, but where traditional restraint was still carried out. Likewise, Laila et al. [41] conducted research into pasung in one city in West Java where the community had access to mental health care. The article found that people still used pasung as the option of care despite more than 2/3 of participants having Indonesian National Insurance, meaning they could use mental health facilities without charge.

Results regarding the geographical spread of pasung use were also contradictory and inconsistent. In many articles, most people in pasung lived in rural areas with poor access to mental health services [4, 7, 47]. One article reported that more than 80% of individuals lived in low socioeconomic and remote areas [54]. In contrast, another study conducted in a rural setting in Singapore by Tay et al. [58] found that pasung could also be found in urban areas with modern infrastructure and accessibility to mental health facilities. Despite these researchers finding only one case in a metropolitan area, they concluded that both rural and urban areas had incidents of pasung. Idaiani and Raflizar [38] also proposed that geographical area and access to mental health service has no relationship with the practice of pasung in Indonesia. Ndetei and Mbwayo [10] furthermore stressed that the practice of chaining could occur globally in more countries than those currently documented.

Besides its influence on the family in conducting pasung, the community also played a role as external support, both for the family and the person in pasung. In one report, neighbours offered casual work for the breadwinner who had a family member in pasung [30]. The neighbourhood provided similar support by facilitating the person to get free insurance and free medication [50].

#### Policy

In addition to individual, interpersonal, and community factors, policy provides a vital context for the practice of pasung and its elimination. The issue of protecting the human rights of PWMD has been a significant concern in many countries, including in Indonesia [16]. However, mental health is not a primary focus compared to physical health in Indonesia. One article reported that in most South-East Asian countries, mental health is not the main priority. As an indicator, the budget spent on mental health is less than 2% of the total health budget, allocated predominantly to psychiatric hospitals. The article also highlighted that there are very few efforts in prevention, promotion, and rehabilitation of mental health. Many mental health programs, including a community-based approach which is mostly concentrated in urban areas, were found to be of poor quality [18].

In LMICs, policy support for mental health problems identified in the literature was inadequate; or, if it existed, the implementation was problematic [8, 18, 43, 45, 46]. While there has been policy prohibiting pasung in Indonesia, there has been a lack of policy, procedure, and funding at the local level. Two studies argued that legislation only is not enough in empowering the protection of human rights for PWMD. Instead, real action from government across central government, the provincial government, and the district level is needed [8, 18]. For instance, a country such as Indonesia already has a Mental Health Act stipulated in 2014, but the regulation at the local level to apply this Act is absent. In contrast to the argument above, Firdaus [34] stated that policy had been developed at a local level in Indonesia and that the issue relates instead to insufficient funding and the implementation of the policy. For instance, in the Mental Health Act and local regulation, it has been stated that pasung perpetrators would be fined or imprisoned [13]; nevertheless, in the implementation, no sanction is given for the perpetrators.

As a result of the complex policy problems, pasung is usually identified as due to the family’s failure to provide appropriate treatment with an expectation that the family know how to manage the PWMD and avoid pasung [8, 62]. In addition, proper policy implementation, including consumer and carer involvement, is yet to occur, with little or no further explanation about how to operationalize this cooperation. In addition to consumer and carer involvement, it has been identified that implementation should be in accordance with the improvement of coordination among all stakeholders, including health professionals, non-health professionals like those in social affairs, not-for-profit organizations, and the community [16, 61].

In Indonesia, the Bebas Pasung Program (Free Pasung Program) involves the provision of community-based mental health services alongside intensive education campaigns [3, 5, 47]. None of the articles measured the effectiveness of the program or how the program is delivered. Different approaches were taken to the delivery of the program in each district or province. For instance, in Bali, Suryani et al. [5] used a community-based program with their “culturally sensitive” intervention which included delivery of clinical interventions (psychiatric medications and counselling) and workshops with families and communities, educating them on mental health issues using respectful language appropriate to their culture and customs. The authors claimed that the model successfully decreased pasung in the Bali region. They measured this based on the in-depth clinical interviews conducted by the psychiatrist with the family and the person using ICD-10 diagnostic criteria. In Jogjakarta, Firdaus [34] studied a community-based service with community volunteers, and Rahman, Marchira and Rahmat [48] added a community nurse to their community-based approach.

In other countries, for example in Somalia [4, 7], the Chain Free Initiative supported by the World Health Organization (WHO) aimed to reduce the number of people restrained in hospital and community settings partly through increasing access to mental healthcare. In a study in China by Guan et al. [2], 271 people with mental illness and restrained at home were identified by the Chinese “686 programs”. After receiving a package of interventions including “unlocking” by a team of mental health professionals, admission to a psychiatric hospital was undertaken, where required, with follow-up by a community mental health team. This study reported that 92% of people remained unrestrained after 7 years of follow-up [2]. However, such an intensive program is not generalizable to countries such as Ethiopia or Indonesia with highly limited mental health resources and where free universal healthcare is not available. Another program reported a social inclusion program and policy which was implemented in East Timor [35]. This program aims to educate the community to enhance community acceptance for persons with a disability, including PWMD. The study claimed that the program increased the opportunity of PWMD to participate in a community activity that increased their well-being.

Despite studies reporting interventions that have been tested and implemented, this review of the literature highlights the lack of an effective intervention to address pasung practice in the community in Indonesia. The existing one (the Free Pasung Program) involves the person being taken to the hospital and being provided with treatment for their mental health over a limited period, and then being returned to the community without further follow-up for the person or support provided to their families and communities. The problems which led to the use of pasung often quickly reappear after hospitalization and the family and community return to using pasung [3, 5].

## Discussion

This systematic review is the first to examine all available peer-reviewed literature to identify the characteristics and the main reasons behind the practice of pasung. This review revealed a limited number of articles available on this topic. Our quality-rating of the included papers revealed that few were of good quality, and many were limited in their discussion and interpretation of findings.

The reviewed studies were mostly conducted in Indonesia with other research among Asian and African Countries. These three countries are among the most populous in the world (China, India, and Indonesia) and three countries are high income countries (Japan, Singapore, and China). In most LMIC across Asia and Africa, mental health care, particularly community mental health care, is scarce. Hence, understanding the reasons behind pasung practice is a profound aspect in developing appropriate programs to reduce pasung.

Analysed through the lens of the SEM, the review identified a range of factors across the individual, interpersonal, community and policy levels, and demonstrates the interconnection of factors within and between these levels. At an individual level, while the diagnosis was unknown for a small number of participants or subjects discussed within the studies, the majority of persons in pasung were diagnosed with a severe mental disorder [7, 41, 47]. People with schizophrenia and with aggressive behaviour were at higher risk compared to those without violent behaviour [41]. They were five times more likely to be at risk of demonstrating violent and aggressive behaviour compared to the general population. This risk increased to 16 times higher when the person had a comorbid condition such as substance misuse [63].

However, a history of mental disorder is not an isolated factor leading to pasung. The review identified further individual-level characteristics including aggressive and violent behaviour, discontinuing medication and past confinement history as being associated with pasung. It was not always clear whether it was the mental illness or aggressive behaviour that is correlated primarily with pasung. The findings support this argument, given that roughly 10% of persons diagnosed with other conditions like dementia, epilepsy, intellectual disability and autism unrelated to mental illness or with comorbidity with mental illness but showed aggressiveness [3, 51]. Thus, pasung could be used for anyone who tends to be violent irrespective of their diagnosis.

We highlight the finding of aggressiveness as it has many forms from verbal, physical, self-injury, or intention to unintentional. Of these, verbal aggression was frequent. In one study, only three out of 15 cases of pasung were due to physical violence; the remainder were categorized as due to past aggressive behaviour with no further explanation [3]. The finding is in line with previous research which described that verbal aggression is common both in acute care or residential settings for PWMD [63]. The number was similar for persons who had dementia [64] and persons with intellectual disability [65]. However, as most of articles were qualitative, it is difficult to determine whether most pasung practice was due to verbal aggressive behaviours. Further research is needed to understand how this type of aggressive behaviour impacts pasung use, and how it might impact the delivery of prevention interventions for pasung.

There are many individual and situational factors for why PWMD may act aggressively [63, 66]. Based on the findings, individual factors leading to pasung were discontinued medication, past confinement history, being predominantly male, single, low educated, and low socioeconomic status [3, 38, 41, 47, 49, 51]. These findings aligned with a previous study which described the risk factors for aggressive behaviour among psychiatric patients, which included being younger age, male, not being married, having previous mental disorder history, and a history of violence or self-destructive behaviour [67]. However, another study by Steinert [68] argued that gender, diagnosis and substance use played a minor role in predicting violence in the community, while the history of previous violence has a significant role. The study also suggested that the role of environmental factors could lead to violence and is mostly underestimated.

Alongside individual level factors, environmental factors are also important. This literature review demonstrated that the family were most likely to use pasung on a family member, usually instigated by the parent or elder sibling [7, 10, 40]. Families believed that the use of pasung was essential to keep the PWMD safe 7, 10, 40]. In addition, the family were faced with a difficult choice between pasung on the one hand and wandering or social nuisance on the other [4, 10]. Therefore, pasung was usually described as a pragmatic action, where the decision to use pasung came after a lengthy discussion with the relatives and community leader. Nevertheless, in the end, the main person responsible for making the decision was the primary caregiver.

However, positioning the problem of pasung on the caregiver within a policy lens, including the decision to put family as the perpetrator in jail, seems unjust. For example, Minas and Diatri [3] reported that, when mental health services are available, the families in their study were willing to unchain the person and accepted all treatment for them when it was offered. Evidently, treatment for people with mental disorders will be optimal if the family are involved in the care and supported to care. The role of the family has proven to be very important in improving medication adherence, preventing recurrence, restoring social roles, and preventing health and economic impacts for caring mentally ill family members [4, 69, 70].

This review also identified the economic strain placed on a family having a family member with a mental illness. This is because the lengthy duration of illness means the PWMD cannot earn an income, exacerbated by the costs of accessing and paying for medications required for treatment. PWMD often require other family members to care for them at home, denying the opportunity for the carer also to have an income [7, 29, 40, 41]. The evidence highlighted that caregivers often take on the burden of caring for the person without help from other relatives and community, meaning it is ultimately their decision whether or not to use pasung.

Another interpersonal factor was mental health literacy; although there was conflicting information on this issue. Many families obtain information about the mental illness and type of treatment, whether medical or alternative, from relatives, neighbours, and the community. While this can be understood as a form of social support to families, the information is sometimes inaccurate, with mental illness often associated with the occult. Two articles [41, 58] argued this contributed to pasung practice, while Asher et al. [7] and Idaiani and Raflizar [38] disagreed. In line with above, Jorm [71] stated that mental health knowledge in the community was often ignored by the public and, as a result, many PWMD were only brought to treatment settings once they were in a chronic state of mental health.

Furthermore, there is a shortfall of interventions to overcome the problem apart from psychoeducation to prevent pasung [32]. Indeed, although most caregivers were poorly educated and sometimes did not gain a formal education, the relationships between low literacy and an increased likelihood that caregivers would use pasung are still debatable. In contrast to physical health, which is observable with signs like pain or swelling, recognition of symptoms of mental disorders is more difficult to understand for the general population. Despite the contrasting arguments, a previous study by Hartini et al. [36] showed that strong literacy, both of individuals and community in terms of mental health knowledge, tended to reduce stigma. Mental health awareness is essential to increase the acceptance of the community, minimize stigmatising beliefs about PWMD, and potentially also to reduce pasung practice.

Family generally behaved and treated the mentally ill family member based on social and cultural norms of the community where they lived. For example, in several studies, the community believed that PWMD were aggressive, dangerous and would not return to ‘normal’ [7, 49, 57, 58], and men were more likely to viewed according to these stigmatised beliefs [59]. Even when no aggressive behaviour was evident, the community perceived the person would behave violently. This negative belief adversely impacted on the person’s ability to cope with the problem and increased their stress. Thus, for security reasons, the community shackled the person [10, 40].

Lack of access to services, particularly in rural areas, was also seen as a significant problem [2, 3, 5, 7]. Despite an assumption that pasung should be less evident in urban areas where mental health facilities are easily accessible, pasung still exists in urban areas [41, 58]. However, most of the articles found that persons in pasung live in a remote area, and mostly low-middle income areas [4, 6–8, 10, 16, 29, 40, 41]. In addition, in many LMICs, psychiatric hospitals are only located in the capital city or more densely populated areas which are often inaccessible for people who live in rural areas, due to problems with transport, time, cost and the capacity of the hospital itself to cater to the needs of these communities.

Furthermore, in Indonesia, for example, nearly 90% of government health funding is also dedicated to psychiatric hospitals, with very limited focus on prevention or early intervention support services in the community [18]. This situation is exacerbated by the limited number and ability of field workers, and the limited number of specialists and treatment facilities. In LMICs, policy support for mental health problems is not adequate and, where it exists, implementation is problematic [8, 18, 43–45]. Another phenomenon that needs attention is the focus on psychiatric hospitals as the main spearhead and waiting for the community to bring people with a mental health condition to these facilities. Inevitably, the mental health service will be inadequate at the primary care level; where it does exist, it is often of low quality. Systems for coordination and follow-up clinical support post-discharge from the hospital settings is also inadequate, creating unnecessary cycles of illness relapse.

The studies identified in this review were predominantly experience-based, focussing on individuals in pasung and their families. However, families are heterogeneous; hence, future research must account for structural, cultural, economic and social variation within the family. For instance, do higher-income families also use pasung? If not, what is the reason? In addition, most of the empirical studies did not consider the pressures placed on families when caring for the person in pasung, such as interpersonal stresses, family burden, finances and other family member needs. Further research could explore these issues in detail. These issues must also be built into support and treatment programs with this population. There is a mismatch between consumer and family preferences and the services offered; what families expect/demand and what is offered by existing support programs. More longitudinal research is needed, ideally connecting multi-disciplinary professionals/stakeholders and those with lived experience at all levels.

Family psychoeducation programs are available, but existing programs vary widely, with inconsistencies in how they are delivered, their content, who conducts them, and where they are conducted (in hospitals or the community). More understanding is needed about the best approaches for delivering psychoeducation to families with lived experience of pasung [55]. The diversity of approaches and the limited strength of evidence found within the studies reviewed here suggest that currently there is no one approach that suits all family situations and that a diversity of approaches is likely to be warranted in order to address pasung. More work is needed in general to develop interventions that address the use of pasung; these interventions need to include family and community participation, and also need to evaluate how they might benefit the consumer themselves. While the need for civic engagement in the development of interventions has been identified, such as inclusion of lived experience perspectives, an understanding of how to do this effectively and how to involve family and the person subjected to pasung remains unclear [5, 8, 16, 55, 61].

### Limitations

Limitations of the studies related to methodological design and the absence of RCTs; although the use of RCTs in pasung is ethically problematic. Most studies using qualitative methods had relatively small samples, unclear or poorly administered analysis methods and an absence of underpinning theory. Therefore, findings cannot be generalized to broader populations or settings. As a result, this review demonstrates that there is no clear evidence to suggest which interventions are best suited to overcome the use of pasung.

This systematic review focused on studies published in English and Indonesian, which may have limited understanding of the findings of studies of pasung reported in other languages. Research predominantly included in this review was undertaken in Indonesia, with a smaller number of studies conducted in other parts of Asia and Africa. In addition, most studies were conducted in LMIC.

## Conclusions

Pasung seems to be the last resort for families of mentally ill family members who show, or are perceived to be at risk of, aggressive and violent behaviour. This situation is exacerbated by limited community care and support, stigma, inaccessibility of mental health services, economic burden and limited regulation of policies that aim to address pasung. The findings highlight the lack of an effective intervention to address pasung practice in the community. While the need for civic engagement and culturally sensitive approaches in the development of community-based interventions, including the inclusion of lived experience perspectives has been identified, an understanding of how to do this effectively, and how to involve family and the person subjected to pasung, remains elusive. A comprehensive understanding is needed to balance the many perspectives at the individual, interpersonal, community and policy level, in order to understand pasung and to inform how to build a more suitable model of care for the person with severe mental illness to reduce the practice of pasung.

## Supplementary information

**Additional file 1: Table S1.** Empirical studies.

**Additional file 2: Table S2.** Reports/discussion papers.

**Additional file 3.** Additional tables.

## Data Availability

All data generated or analysed during this study are included in the published article and its additional files.

## Abbreviations

CRPD: Convention on the rights of people with disabilities
LMIC: Low and middle income countries
PWMD: Person/s with mental disorder
RCT: Randomized controlled trial
SEM: Socio-ecological model
WHO: World Health Organization
