# Supplementary material for: The use of pasung for people with mental illness: a systematic review and narrative synthesis
Source: Int J Ment Health Syst. 2020 Dec 7;14:90. doi: 10.1186/s13033-020-00424-0 (PMC7720453; doi:10.1186/s13033-020-00424-0)
Supplement: Supplementary file 1 — Additional file 1: Table S1. Empirical studies. [file 13033_2020_424_MOESM1_ESM.docx]

**Additional File**

**Table S1:** Empirical Studies

| **No** | **Author, Year & Country** | **Aim & methodology** | **Sampling & participant/study characteristics** | **Data collection & Analysis methods** | **Limitations** |
| --- | --- | --- | --- | --- | --- |
| 1 | Asher, Fekadu, Teferra, De Silva, Pathare & Hanlon  2017  Ethiopia [7] | To understand the experiences of, and reasons for, restraint of people with schizophrenia in community settings in rural Ethiopia in order to develop constructive and scalable interventions.   Qualitative | A purposive sample of people with schizophrenia, their caregivers, community leaders and primary and community health workers in rural Ethiopia. Recruited through district health organisations, community advisory boards, and psychiatric outpatient clinics. | 15 in-depth interviews and 5 focus group discussions (n=35 participants)  Thematic analysis | 1. This was a small sample that may not shed light on the full range of experiences of restraint in terms of duration, pattern or reasons. 2. Understanding restraint was not the primary aim of data collection; one person with schizophrenia had no personal experience of restraint.  3. There may have been social desirability in all types of participants against discussing overtly abusive practices, neglect or long-term restraint 4. Whilst there was a clear picture of distress experienced by family members involved in restraining, there was little data on how restrained person thinks or feels.  5. Little sense of impact of pasung on relationships between the person and caregivers.  6. Despite an equal split of men and women with schizophrenia in this study, due to the small sample size it was difficult to draw conclusions on the impact of gender on patterns and reasons for restraint (suggested that women were more hidden, not visible to researchers). |
| 2 | Broch  2001  Indonesia [6] | To provide an account of the diverse interpretations and explanations for the use of pasung, given by the villagers / cultural ‘insiders’. To examine how ‘craziness’ is conceptualized and experienced in one small Indonesian community. To explore how cultural models and practices shape the social response to psychotic behaviour.  Ethnography | Descriptive, person-centered, ethnographic account of community behaviour and cultural beliefs towards to 'Belo' (person with psychotic behaviour) in Banggai Island, Indonesia. | 2 periods of fieldwork (not specified, though apparent that it was several weeks) to observe villagers' reactions to a young man whom they labelled as 'seriously crazy', and his reactions to them.  Unspecified | Unspecified |
| 3 | Buanasari, Daulima & Wardani  2018  Indonesia [29] | To explore the experience of adolescents having mentally ill parents with pasung.  Qualitative phenomenological study | Purposive sampling. 6 Teenager 12-19 yo who had a parent with mental illness with pasung. Recruited via pasung data at one referral hospital. | In-depth interviews  Colaizzi method noted but not explained. | Parents had been pasung several years ago and two participants didn’t live with their parent after they were in pasung; they were placed with other family. These conditions affected the richness of the data. |
| 4 | Daulima, Nova Helena & Wardani  2019  Indonesia [30] | The study aimed to describe the experience of them breadwinner of family post-pasung to supply the economic needs of the family.   This qualitative descriptive phenomenological | Purposive sampling with 6 key bread winner (1 female and 5 male) who have on of their family member in pasung. The sample taken from the registry of inpatient of local psychiatriic hospital. The patient recently came out from hospital not more than 3 months from the discharged date. | In-depth interview  Colaizzi method | Unspecified |
| 5 | Daulima  2018  Indonesia [31] | To identify and explore the means of preventing mentally ill patients in the community from being subjected to pasung through a test of Daulima’s pasung Decision Questionnaire that measures a family’s intention to use pasung. To test the content validity and split-half reliability of the questionnaire.   Quantitative survey validation study | Purposive sampling. 300 family members of people with mental illness from 5 provinces in Indonesia: West Sumatra, East Kalimantan, West Nusa Tenggara, West Java and the Special Capital Region of Jakarta. Recruited through outpatient clinics at the psychiatric hospitals associated with each province. | Survey-questionaire  1. Split-half realibility test using the Spearman-Brown prediction formula.  2. Content validity test by checking the measurement instrument with experts in the field of psychiatric nursing. | Unspecified |
| 6 | Dewi, Daulima & Wardani  2019  Indonesia [32] | To measure the effects of providing family psychoeducation (FPE) therapy and care decisions without pasung (CDWP) to families of mentally ill patients.   Quantitative - Quasi-experimental pretest-posttest and control group design | 72 families divided into two groups (intervention and control) using purposive sampling. | Zarit Burden Interview qustionnaire measuring 3 dimensions: burden, rejection and incapability.  Chi-squared test, Independent pre-post test and Cronbach's alpha reliability test. | Future research should examine this combination therapy with the addition of individual therapy fr the patient. |
| 7 | Firdaus  2016  Indonesia [34] | to find out the fulfillment of right on health of people with schizophrenia, identified the obstacles and identified the types of human rights abuses faced by them  Qualitative | Government (health agency, social agency, Psychiatric hospital, General Hospital, Police) Patient and their family Non-government organization Academician | Literature search In-depth interview Observation  Unspecified | Unspecified |
| 8 | Guan, Liu, Wu, Chen, Wang, Ma, Wang, Good, Ma, Yu & Good  2015  China [2] | To measure the effectiveness and sustainability of the “unlocking and treatment” intervention and its impact on the well-being of patients’ families.   Pre-post study | Mentally ill patients who were locked at home (in pasung), unlocked as part of “The 686 Program” which involved provision of treatment (hospitalisation where needed, medication - costs covered by the program) and monthly community follow-up by '686' case managers. By 2005, this involved 60 demonstration sites across China and by 2009 161,800 patients were registered, with 42,400 patients receiving regular follow-up. In Stage One of the study, 266 patients unlocked in 2005 were recruited in 2009 using purposive sampling. In 2012, 230 of the 266 cases were re-interviewed (the Stage Two study). | Clinical condition before unlocking and after unlocking using Brief Psychiatric Rating Scale (BPRS) and global severity was assessed with the Clinical Global Impression (CGI) scale.  Comparison of T1 and T2 using the Wilcoxon signed rank test. Chi-square tests to measure male/female and urban/rural differences. | The study was designed after the program was underway; that is, it was not a prospective study with an experimental design. Family burden ratings at T0 were obtained by family care-givers’ retrospective reflections, which may introduce bias. 96% of the sample was people with schizophrenia; therefore, the findings may not be generalization to people with other serious mental illnesses. In a few cases, patients and families refused to be enrolled by the program. |
| 9 | Hall, Kakuma, Palmer, Minas, Martins & Kermode  2019  Timor Leste [35] | To investigate the social experiences of people with mental illness and their families and inform ongoing attempts to improve responses to mental health at the community and service system levels in Timor Leste.  Qualitative | Purposive sample of 85 participants: people with mental illness and their families (n=30), mental health and social service providers (n=23), government decision-makers (n=10), civil society members (n=9), and other community members and organisations (n=13). Recruited through government health services and NGO services in Dili. | In-depth interviews  Framework analysis | There is a risk that participants did not disclose relevant negative experiences, especially to a foreign interviewer. Could not explore the effects of illness type on social exclusion as did not undertake clinical assessment of participants. Sample was not representative and was unlikely to encompass the most vulnerable people with mental illness because the researchers recruited people in contact with health and social services. |
| 10 | Hartini, Fardana, Ariana & Wardana  2018  Indonesia [36] | To explore the correlation between knowledge about mental health and stigma towards people with mental disorders and to find whether there is any marked difference in stigma according to a range of sociodemographic variables (age, sex, marital status, monthly income, education, experience of contact, history of mental disorder, and attitude toward pasung).   Quantitative survey study | Using accidental sampling, 1,269 respondents were recruited from the East Java community via door-to-door community contact. The respondents ranged from 10 to 75 years of age (mean 23.3 yrs), 35% were male; 81.6% were single; , the majority has low educational attainment and low income (deemed to be representative of Indonesian population). | The instruments used were Community Attitudes towards Mental Illness (CAMI), Mental Health Knowledge Schedule (MAKS), and a sociodemographic questionnaire.  Descriptive correlation (unspecified) Score difference (t score or regression) | The study used translated versions of scales developed in Western culture which had not previously been used for general samples in Indonesia. It used self-report measurement. |
| 11 | Nova Helena, Daulima & Wardani  2018  Indonesia [37] | To describe the experience of people with mental disorders, post-pasung, in adapting to society.  Qualitative phenomenological study | Purposive sample of 7 people with mental disorder, with criteria of having been in pasung for a minimum period of three months after hospitalization for the mental disorder and never having experienced a relapse of mental disorder; being able to express their experience; and being aged between 18 and 60 years. | In-depth interviews  Colaizzi method (detail not provided) | Unspecified |
| 12 | Idaiani & Raflizar  2015  Indonesia [38] | To investigate the most dominant factors contributing to pasung practice in Indonesia and an overview of their family characteristics.   Quantitative/survey | Secondary data on basic health research conducted in 2013 and focused on pasung and their relevancies with other characteristics | Document analysis  Unspecified | Unspecified |
| 13 | Irmansyah, Prasetyo & Minas  2009  Indonesia [8] | To examine the legal framework for protection of human rights of persons with mental disorder and the extent to which Indonesia's international obligations concerning the right to health are being met.  Qualitative/Document analysis | Indonesian constitution, Indonesian laws relevant to the right to health, the structure and operation of the National Human Rights Commission, and what is known about violations of the human rights of persons with mental illness from research (largely unpublished, in theses, and in academic institution reports of national scientific meetings), and in local media reports of human rights violations experienced by people with mental illness. | Policy and legal documents analysis, in addition to review of research and media sources for analysis.  Content analysis | Unspecified |
| 14 | Katuuk, Daulima & Wardani  2019  Indonesia [9] | To determine the experience of mental health families in treating the patient using repeated pasung   qualitative/phenomology | 10 participants consisted of family as the main caregiver people subjected to pasung for at least two times in four regencies at North Sulawesi  Purposive sampling | In-depth interviews  Colaizzi method | The family member caregiver can change, which impacted the validity regarding the data. |
| 15 | Laila, Mahkota, Krianto & Shivalli  2018  Indonesia [40] | To explore the perceptions of family members of patients of schizophrenia and other key stakeholders concerning pasung in Bogor Regency, West Java Province in 2017. Qualitative study | Purposive sampling: 12 key stakeholders including family members, neighbors, community leaders, and mental health officers. In-depth interviews were conducted with family members (n = 3) who practiced pasung for patients with schizophrenia and key informant interviews of neighbors, community leaders (two household heads and one from a health cadre) (n = 3) and mental health officers of puskesmas (three midwives). | In-depth interviews  Content analysis | Small and heterogeneous sample |
| 16 | Laila, Mahkota, Shivalli, Bantas & Krianto  2019  Indonesia [41] | To determine the factors associated with pasung among patients with schizophrenia in Bogor Regency, West Java Province, Indonesia in 2017.   Case-control study | Multi-stage sampling to select case and control subject from the registered reports of the Bogor Regency Health Service: 34 sub-districts and 59 health centres; 114 cases (ever subjected to pasung) and 136 control (never subjected to pasung) subjects. Simple random sampling used in each centre until required sample reached for case and control groups. | Semi-structure questionnaire administered to a family member: demographic details plus two 20-item questionnaires devised from a range of sources, focused on 1. knowledge of schizophrenia, and 2. attitudes of the family towards the person.  Descriptive statistics, Chi-square test and multivariate logistic regression analysis | Selection bias could occur when eligible subjects were not willing to participate. Potential for recall bias. |
| 17 | Minas & Diatri  2008  Indonesia [3] | To investigate the nature of restraint and confinement (pasung), the clinical characteristics of people restrained, and the reasons given by families and communities for applying such restraint.  Cross-sectional observational study | Case finding through discussion with doctors and other clinical staff in the 11 puskesmas, visiting villages and having discussions with village heads and other key members of village communities. Interviews with the person who was restrained, with members of the family when they were available, and other members of the village communities. | Interviews and observations during 6 months of working as a psychiatrist visiting a remote district (Samosir)  Unspecified - summary description of variables for 15 cases presented in a table | Unspecified |
| 18 | Nurjannah, Mills, Park & Usher  2015  Indonesia [16] | To understand the provision of mental health care in Indonesia, thereby identifying ways to improve care and better support carers.  Qualitative study | Purposive sampling then theoretical sampling: health professionals, non-health professionals and individuals living with a mental disorder who were well at the time (n = 49). Speciﬁcally, health professionals were nurses (n = 32), general practitioners (n = 1), psychiatrists (n = 1), social workers (n = 4) and psychologists (n = 2). Non-health professionals included family members (n = 3), cadres (n = 4) (mental health volunteers) and community leaders (n = 2), and individuals living with a mental illness who were well at the time (n=2). Recruited from provinces of Jogjakarta and Central Java. Nurses in the initial phase identified further participants from other groups. | In-depth interviews  Grounded Theory | Generalizability, given the research was conducted in only two provinces of Indonesia; that is, it may not take account of the vast cultural diversity in Indonesia or differences across its mental healthcare system. |
| 19 | Puteh, Marthoenis & Minas  2011  Indonesia [47] | To report the findings of a preliminary investigation of the demographic and clinical characteristics of patients who have been admitted to the Banda Aceh Mental Hospital as part of the Aceh Free pasung program.  Cross-sectional descriptive study | 59 (63.4% of all 93 ex-pasung patients admitted, 34 discharged) were inpatients during the data collection period. Patients, ward nurses, and family members who were available at the hospital for interview or could be contacted by telephone. | Interview in order to gain information about variables in scope General medical examination  Clinical diagnostic interview secondary sources (medical file)  Unspecified. Data appeared as descriptive statistical only (counts and percentages) across the range of variables in scope. | Unable to make any judgment about effectiveness of the Aceh Free pasung program at this stage. Preliminary descriptive study only. |
| 20 | Rahman, Marchira & Rahmat  2016  Indonesia [48] | To ​describe the mental health nurse’s role and motivation for implementation of a ‘restraint free’ program at community health centers (Mataram, West Nusa, Tenggara)  Qualitative/Case study design | Purposive sampling  10 mental health nurses certified in basic mental health nursing, 3 caregivers of persons in pasung, 2 community mental health service staff from health municipal of Mataram, 1 community mental health volunteer and 1 person with previous experience of pasung. | In-depth interviews Focus groups Observation  Unspecified | Unspecified |
| 21 | Helena, Daulima & Wardani  2018  Indonesia [49] | to describe the experiences of people with mental illness in living post-pasung life without the support of a spouse  Qualitative/phenomenology | Purposive sampling  4 post pasung treatment from the hospital were recruited. Ages were range between 36-29 years old, had been subjected to pasung up to 3 years and had been discharged from the hospital from 1-4 years. | Indepth interviews Observation Field Notes  Colaizzi method | Unspecified |
| 22 | Read, Adiibokah & Nyame  2009  Ghana [4] | To consider the challenges facing the protection of the human rights of people with mental illness drawing on the results of ethnographic research in Kintampo, a rural community in Ghana, West Africa.   Ethnography informed by transcultural psychiatry (mental illness and a function of the unique experience of being a member of a particular society) | Purposive sample of 40 households with a family member with mental illness, recruited at churches, prayer camps, shrines, family homes, hospitals and clinics. Total of 67 interviews: patients (n=25), carers (n=31), traditional healers (n=3), pastors (n=4), malams (n=1), and imams (n=3). Total of 7 focus groups (n=47) included nurses, young people, cannabis users, church membes and parents. | Ethnographic methods included observation, conversation, semi-structured interviews and focus group discussions with people with mental illness, carers, healers, health workers and community members.  Grounded theory | Small sample size and particular personal, historical, social and cultural factors varied; therefore, findings not generalizable to other areas. Potential impact of researchers: the presence of both educated Ghanaian researchers and a white European researcher undoubtedly influenced the responses (eg. informants may have been able to say things to a 'stranger' that they could not say to a member of the community). The use of Twi language may have disadvantaged those for whom it was not their first language; the process of translation may have led to some loss or distortion of meaning. |
| 23 | Reknoningsih, Daulima & Putri  2014  Indonesia [50] | To elaborate the family's experience of caring for a person, with seclusion or restraint, using a phenomenological approach.  Qualitative | Purposive sampling  7 caregivers (30-59 years old) who had their family member in pasung | Indepth interviews observation  Cresswell analyzing steps | Unspecified |
| 24 | Riany, Cuskelly & Meredith  2016  Indonesia [51] | To investigate how Indonesian mothers from a range of backgrounds and without a child with autism understood autism and the most appropriate ways to parent such a child.  Qualitative | Purposive sample of 9 Indonesian mothers aged 30 to 69 years (Mean=50, SD = 13.44) (mothers and grandmothers of children aged 3–10 years with no disabilities). Mothers were selected because of their substantial authority within the Indonesian family and major responsibility in childrearing activities. Recruited through discussion with a local government employee. | Semi-structured interviews  Thematic analysis | Small sample size. More heterogeneous sample needed, as only represented 4 of the ethnicities, and 3 of the 6 religions in Indonesia. Did not elicit rural perspectives. Research perception as 'expert' may have influenced responses. Potentially leading interview questions and need for more open questions. |
| 25 | Suharto  2014  Indonesia [54] | To describe the pasung condition, self-care level, social and legal aspects of pasung patients.  Quantitative | 28 families comprising 25 ex pasung and 3 where their family member was currently in pasung.   Total sampling | Unspecified  Unspecified | Unspecified |
| 26 | Suhron  2017  Indonesia [55] | To analyze the ability of families to care for people with mental disorder in pasung before and after family psychoeducation delivered in Healthy Foundation Bani Amrini Tanah Merah District.  Quasi-experiment | 30 caregivers who came to the inpatient unit at Yayasan Sehat who accompanied patient diagnosed with a severe mental disorder  Sampling technique unspecified | Pre and post questionaires were given to measure the knowledge of the respondents.  Wilcoxson test | Unspecified |
| 27 | Suhron, Yusuf & Subarniati  2018  Indonesia [56] | To assess the potential of increasing family ability in managing post-restraint schizophrenia patient  Observational study | 157 families who have a family member with severe mentally ill from 6 districts in East Java  Cluster sampling | Questionnaire  Descriptive analysis SEM | Unspecified |
| 28 | Suryani, Lesmana & Tiliopoulos  2011  Indonesia [5] | To identify, map and treat the clinical features of mentally ill people who had been isolated and restrained by family and community members in Karangasem regency of Bali, Indonesia, and to evaluate the outcomes of this intervention.  Epidemiological Survey Study + Intervention (medication, counselling and education) | A 10-month epidemiological population survey was carried out in Karangasem regency of Bali, Indonesia. A total of 404,591 individuals were clinically interviewed, of which 895 individuals with mental health problems were identiﬁed, with 23 satisfying criteria of physical restraint and conﬁnement. Of the latter, twenty were males; age range was 19–69 years, all diagnosed with schizophrenia-spectrum disorder (ICD-10 diagnostic criteria). Duration of restraint ranged from 3 months to 30 years (mean = 8.1 years, SD = 8.3 years). | Survey, In-depth interview, (with family consent) combination of medication, counselling and education, with each patient being followed up, initially on a weekly basis and for over a year, or until they reached a functional state.  Unspecified | Unspecified |
| 29 | Tanaka, Tuliao, Tanaka, Yamashita & Matsuo  2018  Philippines [57] | To investigate the experiences of being stigmatised for people with mental health problems in the Philippines.   Qualitative (Constructivist grounded theory approach) | Purposive sample in an urban city (Muntinlupa)of people with mental health problems (n=39), their carers (n=39)(20 interviews as a dyad and 19 interviews with the carer only due to person experiencing communication difficulties), community health volunteers in charge of the district in which the person lived (n=11), in addition to interviews with government health workers (n=7; 3 community health volunteers, 2 nurses, a doctor and 1 rehab program volunteer) and observation and discussions with 85 staff at the health service. People with mental health problems and carers were recruited in cooperation with community health volunteers and from the outpatient clinical of a psychiatrist. | Semi-structured in-depth interviews 1-month of observations and discussions with staff at community health services.  Grounded theory | Did not include people with common mental health problems who were not using psychiatric services.  Additionally, cultural and language barriers may have played a part in data collection and interpretation. Some interviews were too short to be considered an in-depth interview. For almost half of participants, the study relied on accounts by others (carers and workers) about the person's experience of stigma. Participants drawn from one city, therefore results may not be generalizable to other areas of the Philippines. |
| 30 | Tay, Chan, Ho & Lal  2017  Singapore [58] | To describe the case of a patient, with schizophrenia who was physically chained by her mother in their house for more a decade, and to exlore the cultural and ethical issues surrounding the case.  Case study | Single case study, a woman 44 years old ﬁrst presented to the mental health services when she was 18 years old, with paranoid delusions, hallucinations and disorganized behaviour. Several relapses in the context of non-compliance, mother's apprehension about western medicine, and suicide of another family member, with consequent increase in mother's mistrust and paranoia, leading to use of pasung for the daughter. | Unspecified - presentation of direct quotes from various family members suggests that they were interviewed by the researcher.  Unspecified | Unspecified |
| 31 | Vijayalakshmi, Reddemma & Math  2012  India [60] | To investigate gender differences in perceived human rights needs at the family and community levels in individuals with mental illness in India.   Descriptive survey study | 100 asymptomatic individuals with mental illness at a tertiary care center. Subject selection employed a random sampling method. | Face-to-face interviews based on a structured needs assessment questionnaire, including socio-demographic data, the Clinical Global Impression-Improvement (CGI-I) Scale, and needs assessment based on the Universal Declaration of Human Rights (physical, emotional, religious social ethical needs) and review of the literature.  Descriptive and inferential statistics (Chi-square tests). Validation of needs questionnaire by a team of 11 experts. | The study population was restricted to people with mental illness currently receiving treatment in an outpatient department at a tertiary centre. |
| 32 | Wirya  2017  Indonesia [61] | To deconstruct the meaning of madness that has been developing in the community and how this discourse of madness shaped crimes for those in pasung  Qualitative/ethnography | 2 families who are the members were in pasung (uce and Dayu) | In-depth interviews  Unspecified | Unspecified |
| 33 | Wulandari, Daulima & Wardani  2019  Indonesia [62] | To identify the resistance against stigma as part of the recovery process in post pasung mentally ill patient  Qualitative/phenomenology | 12 Post pasung mentally ill person age 28-51 years old. They were treated in hospital and has ongoing treatment at outpatient and rehabilitation unit at the Sangli psychiatric hospital, Bali. The patient has been assesed by the psychiatrist with Postive negative syndrome scale with the score ≤ 60.   Purposive sampling | Indepth interviews  Colaizzi method | Unspecified |
| 34 | Yusuf & Tristiana  2018  Indonesia [10] | To describe family support on post pasung patients  Qualitative/phenomenology | 9 families who have ex pasung patient  purposive sampling | Indepth interviews  Colaizzi method | Unspecified |

**Supplementary Files**

**Table 2: Reports/Discussion Papers**

| **No** | **Author, Year & Country** | **Main Purpose (Who and/or what is involved?)** | **How is the problem of pasung defined?** | **Who are they talking about?** | **Proposed solutions** |
| --- | --- | --- | --- | --- | --- |
| 1 | Alem  2000  Ethopia [27] | To describe the condition of human rights and psychiatric care in an African country in reference to Ethiopia. | People in most African countries believe that mental illness is caused by supernatural evil spirits. The method used to cure the disease is predominantly by traditional or spritual healers. After trying traditonal healers, families usually keep the patient at home under restraint until they are no longer aggressive. | Human rights and psychiatric care in Africa in reference to the Ethiopian community. | Strengthen the economy to help address the basic needs for survival of its citizens. Provide more psychiatric hospitals and train more psychiatrists and nurses; address the shortage of appropriate medications; create mental health legislation; build social support systems; develop forensic services to provide more appropriate care settings. |
| 2 | Anto & Colucci  2015  Indonesia [28] | 1. To describe a pasung condition from a lived experience perspective through painting, narration and poetry.  2. To understand pasung and the reasons behind the practice of pasung. | Pasung is an Indonesian term for a physical restraint using wooden stocks, chains, cages, shackles, rope or locking the person in an isolated place. These methods are applied to a person who is deemed to be abnormal. In many cases, the perpetrators were families, with some instances in which pasung was imposed by the community leader. | Single case study of pasung from the perspective of lived experience (from a person who has been subjected to pasung) | 1. Intensive education campaigns 2. Community based initiatives 3. New investment in mental health |
| 3 | Eka & Daulima  2019  Indonesia [33] | To explain the factor related to pasung on people with mental illness from 5 databses i.e. Science direct, Proquest, Scopus, Ebsco, and Google Scholar. | The practice of pasung on mentally ill person influenced by three main factor 1. The patient condition e.g. aggressive behaviour, wandering, violent behaviour, medication drop out. 2. The factor from relatives including burn out, mental health illiteracy, financial issues, helplessness and the failure of alternative treatment. The final decision to implement pasung was discussed among family member, considerating community voice.  3. Factors from community e.g. stigma and discrimination. The community has a profound influence on the family’s use of pasung. | Factor related to pasung which is commonly found in developing countries | 1. Educating the person about medication compliance. 2. Family education about medication compliance to overcome stigma about its use. 3. Community empowerment; in particular, empowerment of community leaders with influence in decision-making (though a limited description of the empowerment process was provided. |
| 4 | Jones, Asare, El Masri, Mohanraj, Sherief & Van Ommeren  2009  Chad [39] | To describe ﬁeld experiences in establishing mental health services in ﬁve humanitarian settings experiencing complex emergencies (eg. mass displacement due to war, conflict, natural disaster): Sierra Leone, Chad, Aceh, North West Frontier Province. | Physical restraint is common because, in the absence of care, families see it as the most humane solution. Some people are chained and abandoned by family who become displaced. Complex emergencies also disrupt essential services within these countries; normal services are disrupted and insufficient to meet the needs of a dislocated population. Long-term chronic conflict, poverty and marginalisation has negatively impacted people with mental illness and contributed to greater prevalence of mental illness. Some countries have been without healthcare for many years. Families hiding sick relatives and disrupted help-seeking are problems exacerbated in humanitarian settings, and emergency care providers often lack appropriate training. | People with severe mental disorders are a neglected and vulnerable group in complex emergencies. | Recommended set of minimum interventions for people with mental illness in humanitarian settings. Appropriate training and supervision of humanitarian workers. More care in mental heath capacity building in primary health care, often over-burdened already. Psychosocial and clinical support need to go hand-in-hand so that people with severe mental illness are not underserved. |
| 5 | Maramis, Van Tuan & Minas  2011  Indonesia [18] | To present an analysis of mental health systems among South Asian Countries. | pasung perceived as a government responsibility involving system reform and service integration across systems: The government of Aceh and the national government of Indonesia have explicitly committed to eradicate restraint and confinement of mentally ill people in the community, the Aceh Free pasung programme. | Resources in mental health systems in Southeast Asian countries. | Service system integration; improving access to primary care; leadership at all levels (especially political leadership); locally developed solutions rather than those simply imported from elsewhere; collaboration among ASEAN countries on development initiatives. |
| 6 | Marthoenis, Yessi, Aichberger & Schouler-Ocak  2016  Indonesia [42] | To understand the mental healthcare system in Aceh Province, Indonesia; in particular, on burden, on the healthcare system, system development, service delivery and cultural issues from the Tsunami in 2004 until the present. | The practice of pasung is common, culturally acceptable, has been existed within community for a long time. It usually happens within the person's local community. It is performed by lay persons related to mental illness, by family or community due to various reasons. In addition, it happens in all provinces in Indonesia; not limited to Aceh.  Pasung has been an enormous issue that makes governments create the goal of being Free from pasung. The treatment by releasing the person from pasung and referral to the mental hospital for limited time. | Mental health system in Aceh Province before and after tsunamy | 1. Stigma reduction 2. Local treatment with cultural approach 3. Mental health service in public health centre 4. Insurance for the people with mental disorder |
| 7 | Miller  2012  Indonesia [43] | To describe an unconventional approach by the Indonesian Government in dealing with access to mental health care, where GPs and community mental health nurses were trained to provide basic consultation and support for people with mental health conditions. | Confinement often arose not out of cruelty but a lack of access to support and care alternatives. | Aceh pasung community mental health program. Specifically in one village in the outskirt of Aceh where some mental health patients were restrained due to lack of mental health services. | Training of general practioners, nurses and volunteers located in regional areas and local villages. |
| 8 | Molodynski, O'Brien & Burns  2017  Low and high income countries [20] | To report the use of physical restraint both in the community and mental health institutions. | pasung is literally translated as to ‘tie’ or ‘bind’. In practice it is physical restraint by these or other means, such as chaining, locking in animal sheds or other highly restrictive measures. It can continue for anything from hours to decades and is typically prolonged. It occurs in hospitals, healing centres and within communities and families. There is evidence that these practices occur in other countries, albeit with varying frequency, level of state involvement and overtness. | Mentally ill patients who experience coersion both in the community and mental health institutions; with comparion within low-middle income countries and high income countries. | No solutions given. |
| 9 | Ndetei & Mbwayo  2010  Africa [14] | To call for closeer auditing to determine how common the practice of pasung is in Africa. | Physical restraints and beatings were the most practiced ways of handling aggressive behaviour by people with mental illness in the communities. Chaining as a physical restraint was a standard practice in the seclusion area and it was often demanded by the relatives who were the only people to nurse such patients. Mistreatment is rarely investigated. pasung is due to lack of knowledge about mental illness. | People with mental illness in the community where families often resort to pasung to manage aggression by family members with mental illness, and in Psychiatric Hospitals in Somalia where physical restraint is also commonly found as a treatment. | Closer monitoring/auditing of the problem and improved education of the community and families. |
| 10 | Patel & Bhui  2018  Ghana [44] | Describes three main issues arising from a study by Ofori-Atta of an RCT conducted in a prayer camp run by faith healers for people in pasung, and discussed concerns about pasung generally:  1. Ethical considerations in clinical trials involving patients in pasung 2. The effect of medication on pasung use 3. The strategy to stop pasung | Chaining is a ‘long-standing custom’ in which residents who are ‘agitated, or considered at high risk for harming self or others, or leaving without informing staff’ are shackled using ‘a chain of approximately two feet in length which was fastened around one leg and anchored to the concrete floor. | Severe mental disorder patient who were chain in rural Ghana | Legislative action accompanied by a concerted effort to provide community-based psychosocial support services focused on social integration. In indonesia, a national campaign to raise mental health awareness and intersectorial collaboration has led to 4200 individuals identified in pasung being freed. Task-sharing of frontline delivery of psychosocial strategies is also an effective and affordable approach. |
| 11 | Patel, Goel & Desai  2009  India [45] | Describes a plan that seeks to integrate the evidence for the treatment of specific mental and neurological disorders based on a task-shifting paradigm, for scaling up services at the level of a defined population. | Family members, left often without any access to care, are forced to rely on restraints and other degrading practices to manage disturbed behaviours of their relatives with mental illness. The vast majority of those who suffer from mental and neurological disorders with no obvious externally apparent symptoms (eg. those with depression or alcohol-use disorders) are simply ignored altogether. | Mental and neurological disorders account for a large, and growing, burden of disease in low- and middle-income countries. Most people do not have access to even basic health care for these disorders. | Succcess through sensible local application of broad principles as per the District Mental Health Plan, primary through upskilling of primary healthcare and volunteers at local level. |
| 12 | Patel, Saxena, Lund, Thornicroft, Baingana, Bolton, Chisholm, Collins, Cooper, Eaton & Herrman  2018  Low-midle income countries [46] | Reassessment of the global mental health agenda in the context of the Sustainable Development Goals as part of coordinated global actions to address mental health. | Across the globe, people living with mental disorders have often been hidden, tortured, abandoned, or left to die. In many countries, lack of access to health services, housing and employment, and sometimes extreme violation of basic rights, is common. The quality of care received by many people, in particular those affected by severe mental disorders and disabilities, was poor in all countries and was often associated with abuses of their fundamental human rights (eg, forced restraints, physical and sexual violence, and torture. | The specific references to mental health and substance use as  targets within the health Sustainable Development Goals | Six key actions required: 1. Scale up of mental health services, and fully integrated; 2. Barriers and threats to mental health need to be addressed through greater mental health promotion and protection; 3. Public policies are needed that engage a wide range of stakeholders within and beyond health (eg. education, workplaces, social welfare, criminal justice) ie. social and environmental determinants of health; 4. Embrace new opportunities to train non-specialists and use digital technology, and mobile the voice of lived experience of mental disorders; 5. Additional investment and redistribution of mental health budgets from large hospitals to community based services; and 6. Investment in research and innovation. |
| 13 | Sa'ad & Bokharey  2001  Pakistan [52] | To describe a mental health service and practice of restraint in one of Pakistan area, Lahore, in mid-1991. | Shortages in trained helth professionals; urbanisation leading to rural shortages; longstanding negative cultural beliefs about mental illness; inhumane practices: Chaining people to trees around holy shrines; to cover the expense, families can become beholden to serve as domestic servants to the shrine managers. | 1. Mental health services in Pakistan  2. People with mental illness chained to trees next to shrines 3. The traditional and spiritual healers and their practices to 'cure' mental illness | Fundraising charity to source medications. Providing psychiatric medications and follow-up monitoring to people in pasung. |
| 14 | Saribu & Napitulu  2009  Indonesia [13] | The aim is to describe The legislation concerning people with mental disorder in Indonesia | pasung is defined as a failure of the law to protect people with a mental disorder who cannot otherwise protect their right due to illness condition.  pasung is torture inflicted on the individual subjected to pasung for the purpose of punishing, intimidation or coercion of the person. | Indonesian legal system, national law which regulates the right of persons with mental disorder i.e.  1. Law No.23 of 1992 concerning health 2. Law No.39 of 1999 concerning human rights 3. Law No.4 of 1997 concerning persons with disabilities 4. Pela code of Indonesia  5. Indonesian criminal procedure code (pasung could be classified as a criminal deed; however, up until now no perpetrator of pasung has been punished). | The government should provide proper mental health treatments for people with mental disorders. Besides that to create understanding among law enforcers, further legal discussions and legal cooperation are also significant and need to be compulsory in order to handle pasung in a comprehensive way. |
| 15 | Stratford, Kusuma, Goding, Paroissien, Brophy, Damayanti, Fraser & Ng  2014  Indonesia [53] | To describe a community-based recovery-oriented practice approach for ex-pasung patients, and to discuss the reasons for why this approach has been successful so far. | Common; a broader community issue; a mismatch in health and social welfare resource allocation in the context of rapid economic growth; a Ministry of Health responsibility to provide proper psychiatric and physical health treatment; Ministry of Social Affairs responsbility to provide social rehabilitation services for people with disabilities and other disadvantage, including homelessness. "Many people living with mental illness in Indonesia are physically restrained at home, some for many years, conﬁned to cages, locked rooms or huts and sometimes in chains or wooden stocks, with no psychiatric treatment and deteriorating physical health. This practice of restraint, known as pasung, is not restricted to Indonesia." | People with mental illness who have been in pasung or experienced homelessness | Access to affordable, ongoing care in the community; A social rehabilitation approach: A staged social rehabilitation program of support provided at a live-in community-based centre over 1-2 years as part of return to home/community. In-reach by psychiatrists and medical staff. Peer support, family re-unification, enhancing the person's ability to contribute to the community (occupation). |
| 16 | Ulya  2019  Indonesia [59] | To discuss the bioethical and health law perspective on the use of pasung for people with mental illness in Indonesia | pasung has become a national problem. Even though Indonesia has released a program aiming to eliminate the use of pasung and established a National Mental Health Act that prohibits the practice of pasung, the practice continues to be a routinely used option in treating people with mental disorder. In addition, the achievement of 'National Free from pasung' is far from reaching its target, which has frustated many people seeking to address human rights for this group in the Indonesian community. | Nioethic, health regulation particularly related with mental health | 1. Indonesian government programs need to be integrated particularly the process of deinstitutionalizing mental health services. 2. Integrating community and policy changes should help make mental health services move towards de-formalization. This process increases the chance of the patient being returned to the community and living in an environment free from coercion.  3. Need a real implementation that ensure patients live in their families and communities free from coercion. |
