# Supplementary material for: The use of pasung for people with mental illness: a systematic review and narrative synthesis
Source: Int J Ment Health Syst. 2020 Dec 7;14:90. doi: 10.1186/s13033-020-00424-0 (PMC7720453; doi:10.1186/s13033-020-00424-0)
Supplement: Supplementary file 2 — Additional file 2: Table S2. Reports/discussion papers. [file 13033_2020_424_MOESM2_ESM.docx]

**Table S2:** Reports/Discussion Papers

| **No** | **Author, Year & Country** | **Main Purpose (Who and/or what is involved?)** | **How is the problem of pasung defined?** | **Who are they talking about?** | **Proposed solutions** |
| --- | --- | --- | --- | --- | --- |
| 1 | Alem  2000  Ethopia [27] | To describe the condition of human rights and psychiatric care in an African country in reference to Ethiopia. | People in most African countries believe that mental illness is caused by supernatural evil spirits. The method used to cure the disease is predominantly by traditional or spritual healers. After trying traditonal healers, families usually keep the patient at home under restraint until they are no longer aggressive. | Human rights and psychiatric care in Africa in reference to the Ethiopian community. | Strengthen the economy to help address the basic needs for survival of its citizens. Provide more psychiatric hospitals and train more psychiatrists and nurses; address the shortage of appropriate medications; create mental health legislation; build social support systems; develop forensic services to provide more appropriate care settings. |
| 2 | Anto & Colucci  2015  Indonesia [28] | 1. To describe a pasung condition from a lived experience perspective through painting, narration and poetry.  2. To understand pasung and the reasons behind the practice of pasung. | Pasung is an Indonesian term for a physical restraint using wooden stocks, chains, cages, shackles, rope or locking the person in an isolated place. These methods are applied to a person who is deemed to be abnormal. In many cases, the perpetrators were families, with some instances in which pasung was imposed by the community leader. | Single case study of pasung from the perspective of lived experience (from a person who has been subjected to pasung) | 1. Intensive education campaigns 2. Community based initiatives 3. New investment in mental health |
| 3 | Eka & Daulima  2019  Indonesia [33] | To explain the factor related to pasung on people with mental illness from 5 databses i.e. Science direct, Proquest, Scopus, Ebsco, and Google Scholar. | The practice of pasung on mentally ill person influenced by three main factor 1. The patient condition e.g. aggressive behaviour, wandering, violent behaviour, medication drop out. 2. The factor from relatives including burn out, mental health illiteracy, financial issues, helplessness and the failure of alternative treatment. The final decision to implement pasung was discussed among family member, considerating community voice.  3. Factors from community e.g. stigma and discrimination. The community has a profound influence on the family’s use of pasung. | Factor related to pasung which is commonly found in developing countries | 1. Educating the person about medication compliance. 2. Family education about medication compliance to overcome stigma about its use. 3. Community empowerment; in particular, empowerment of community leaders with influence in decision-making (though a limited description of the empowerment process was provided. |
| 4 | Jones, Asare, El Masri, Mohanraj, Sherief & Van Ommeren  2009  Chad [39] | To describe ﬁeld experiences in establishing mental health services in ﬁve humanitarian settings experiencing complex emergencies (eg. mass displacement due to war, conflict, natural disaster): Sierra Leone, Chad, Aceh, North West Frontier Province. | Physical restraint is common because, in the absence of care, families see it as the most humane solution. Some people are chained and abandoned by family who become displaced. Complex emergencies also disrupt essential services within these countries; normal services are disrupted and insufficient to meet the needs of a dislocated population. Long-term chronic conflict, poverty and marginalisation has negatively impacted people with mental illness and contributed to greater prevalence of mental illness. Some countries have been without healthcare for many years. Families hiding sick relatives and disrupted help-seeking are problems exacerbated in humanitarian settings, and emergency care providers often lack appropriate training. | People with severe mental disorders are a neglected and vulnerable group in complex emergencies. | Recommended set of minimum interventions for people with mental illness in humanitarian settings. Appropriate training and supervision of humanitarian workers. More care in mental heath capacity building in primary health care, often over-burdened already. Psychosocial and clinical support need to go hand-in-hand so that people with severe mental illness are not underserved. |
| 5 | Maramis, Van Tuan & Minas  2011  Indonesia [18] | To present an analysis of mental health systems among South Asian Countries. | pasung perceived as a government responsibility involving system reform and service integration across systems: The government of Aceh and the national government of Indonesia have explicitly committed to eradicate restraint and confinement of mentally ill people in the community, the Aceh Free pasung programme. | Resources in mental health systems in Southeast Asian countries. | Service system integration; improving access to primary care; leadership at all levels (especially political leadership); locally developed solutions rather than those simply imported from elsewhere; collaboration among ASEAN countries on development initiatives. |
| 6 | Marthoenis, Yessi, Aichberger & Schouler-Ocak  2016  Indonesia [42] | To understand the mental healthcare system in Aceh Province, Indonesia; in particular, on burden, on the healthcare system, system development, service delivery and cultural issues from the Tsunami in 2004 until the present. | The practice of pasung is common, culturally acceptable, has been existed within community for a long time. It usually happens within the person's local community. It is performed by lay persons related to mental illness, by family or community due to various reasons. In addition, it happens in all provinces in Indonesia; not limited to Aceh.  Pasung has been an enormous issue that makes governments create the goal of being Free from pasung. The treatment by releasing the person from pasung and referral to the mental hospital for limited time. | Mental health system in Aceh Province before and after tsunamy | 1. Stigma reduction 2. Local treatment with cultural approach 3. Mental health service in public health centre 4. Insurance for the people with mental disorder |
| 7 | Miller  2012  Indonesia [43] | To describe an unconventional approach by the Indonesian Government in dealing with access to mental health care, where GPs and community mental health nurses were trained to provide basic consultation and support for people with mental health conditions. | Confinement often arose not out of cruelty but a lack of access to support and care alternatives. | Aceh pasung community mental health program. Specifically in one village in the outskirt of Aceh where some mental health patients were restrained due to lack of mental health services. | Training of general practioners, nurses and volunteers located in regional areas and local villages. |
| 8 | Molodynski, O'Brien & Burns  2017  Low and high income countries [20] | To report the use of physical restraint both in the community and mental health institutions. | pasung is literally translated as to ‘tie’ or ‘bind’. In practice it is physical restraint by these or other means, such as chaining, locking in animal sheds or other highly restrictive measures. It can continue for anything from hours to decades and is typically prolonged. It occurs in hospitals, healing centres and within communities and families. There is evidence that these practices occur in other countries, albeit with varying frequency, level of state involvement and overtness. | Mentally ill patients who experience coersion both in the community and mental health institutions; with comparion within low-middle income countries and high income countries. | No solutions given. |
| 9 | Ndetei & Mbwayo  2010  Africa [14] | To call for closeer auditing to determine how common the practice of pasung is in Africa. | Physical restraints and beatings were the most practiced ways of handling aggressive behaviour by people with mental illness in the communities. Chaining as a physical restraint was a standard practice in the seclusion area and it was often demanded by the relatives who were the only people to nurse such patients. Mistreatment is rarely investigated. pasung is due to lack of knowledge about mental illness. | People with mental illness in the community where families often resort to pasung to manage aggression by family members with mental illness, and in Psychiatric Hospitals in Somalia where physical restraint is also commonly found as a treatment. | Closer monitoring/auditing of the problem and improved education of the community and families. |
| 10 | Patel & Bhui  2018  Ghana [44] | Describes three main issues arising from a study by Ofori-Atta of an RCT conducted in a prayer camp run by faith healers for people in pasung, and discussed concerns about pasung generally:  1. Ethical considerations in clinical trials involving patients in pasung 2. The effect of medication on pasung use 3. The strategy to stop pasung | Chaining is a ‘long-standing custom’ in which residents who are ‘agitated, or considered at high risk for harming self or others, or leaving without informing staff’ are shackled using ‘a chain of approximately two feet in length which was fastened around one leg and anchored to the concrete floor. | Severe mental disorder patient who were chain in rural Ghana | Legislative action accompanied by a concerted effort to provide community-based psychosocial support services focused on social integration. In indonesia, a national campaign to raise mental health awareness and intersectorial collaboration has led to 4200 individuals identified in pasung being freed. Task-sharing of frontline delivery of psychosocial strategies is also an effective and affordable approach. |
| 11 | Patel, Goel & Desai  2009  India [45] | Describes a plan that seeks to integrate the evidence for the treatment of specific mental and neurological disorders based on a task-shifting paradigm, for scaling up services at the level of a defined population. | Family members, left often without any access to care, are forced to rely on restraints and other degrading practices to manage disturbed behaviours of their relatives with mental illness. The vast majority of those who suffer from mental and neurological disorders with no obvious externally apparent symptoms (eg. those with depression or alcohol-use disorders) are simply ignored altogether. | Mental and neurological disorders account for a large, and growing, burden of disease in low- and middle-income countries. Most people do not have access to even basic health care for these disorders. | Succcess through sensible local application of broad principles as per the District Mental Health Plan, primary through upskilling of primary healthcare and volunteers at local level. |
| 12 | Patel, Saxena, Lund, Thornicroft, Baingana, Bolton, Chisholm, Collins, Cooper, Eaton & Herrman  2018  Low-midle income countries [46] | Reassessment of the global mental health agenda in the context of the Sustainable Development Goals as part of coordinated global actions to address mental health. | Across the globe, people living with mental disorders have often been hidden, tortured, abandoned, or left to die. In many countries, lack of access to health services, housing and employment, and sometimes extreme violation of basic rights, is common. The quality of care received by many people, in particular those affected by severe mental disorders and disabilities, was poor in all countries and was often associated with abuses of their fundamental human rights (eg, forced restraints, physical and sexual violence, and torture. | The specific references to mental health and substance use as  targets within the health Sustainable Development Goals | Six key actions required: 1. Scale up of mental health services, and fully integrated; 2. Barriers and threats to mental health need to be addressed through greater mental health promotion and protection; 3. Public policies are needed that engage a wide range of stakeholders within and beyond health (eg. education, workplaces, social welfare, criminal justice) ie. social and environmental determinants of health; 4. Embrace new opportunities to train non-specialists and use digital technology, and mobile the voice of lived experience of mental disorders; 5. Additional investment and redistribution of mental health budgets from large hospitals to community based services; and 6. Investment in research and innovation. |
| 13 | Sa'ad & Bokharey  2001  Pakistan [52] | To describe a mental health service and practice of restraint in one of Pakistan area, Lahore, in mid-1991. | Shortages in trained helth professionals; urbanisation leading to rural shortages; longstanding negative cultural beliefs about mental illness; inhumane practices: Chaining people to trees around holy shrines; to cover the expense, families can become beholden to serve as domestic servants to the shrine managers. | 1. Mental health services in Pakistan  2. People with mental illness chained to trees next to shrines 3. The traditional and spiritual healers and their practices to 'cure' mental illness | Fundraising charity to source medications. Providing psychiatric medications and follow-up monitoring to people in pasung. |
| 14 | Saribu & Napitulu  2009  Indonesia [13] | The aim is to describe The legislation concerning people with mental disorder in Indonesia | pasung is defined as a failure of the law to protect people with a mental disorder who cannot otherwise protect their right due to illness condition.  pasung is torture inflicted on the individual subjected to pasung for the purpose of punishing, intimidation or coercion of the person. | Indonesian legal system, national law which regulates the right of persons with mental disorder i.e.  1. Law No.23 of 1992 concerning health 2. Law No.39 of 1999 concerning human rights 3. Law No.4 of 1997 concerning persons with disabilities 4. Pela code of Indonesia  5. Indonesian criminal procedure code (pasung could be classified as a criminal deed; however, up until now no perpetrator of pasung has been punished). | The government should provide proper mental health treatments for people with mental disorders. Besides that to create understanding among law enforcers, further legal discussions and legal cooperation are also significant and need to be compulsory in order to handle pasung in a comprehensive way. |
| 15 | Stratford, Kusuma, Goding, Paroissien, Brophy, Damayanti, Fraser & Ng  2014  Indonesia [53] | To describe a community-based recovery-oriented practice approach for ex-pasung patients, and to discuss the reasons for why this approach has been successful so far. | Common; a broader community issue; a mismatch in health and social welfare resource allocation in the context of rapid economic growth; a Ministry of Health responsibility to provide proper psychiatric and physical health treatment; Ministry of Social Affairs responsbility to provide social rehabilitation services for people with disabilities and other disadvantage, including homelessness. "Many people living with mental illness in Indonesia are physically restrained at home, some for many years, conﬁned to cages, locked rooms or huts and sometimes in chains or wooden stocks, with no psychiatric treatment and deteriorating physical health. This practice of restraint, known as pasung, is not restricted to Indonesia." | People with mental illness who have been in pasung or experienced homelessness | Access to affordable, ongoing care in the community; A social rehabilitation approach: A staged social rehabilitation program of support provided at a live-in community-based centre over 1-2 years as part of return to home/community. In-reach by psychiatrists and medical staff. Peer support, family re-unification, enhancing the person's ability to contribute to the community (occupation). |
| 16 | Ulya  2019  Indonesia [59] | To discuss the bioethical and health law perspective on the use of pasung for people with mental illness in Indonesia | pasung has become a national problem. Even though Indonesia has released a program aiming to eliminate the use of pasung and established a National Mental Health Act that prohibits the practice of pasung, the practice continues to be a routinely used option in treating people with mental disorder. In addition, the achievement of 'National Free from pasung' is far from reaching its target, which has frustated many people seeking to address human rights for this group in the Indonesian community. | Nioethic, health regulation particularly related with mental health | 1. Indonesian government programs need to be integrated particularly the process of deinstitutionalizing mental health services. 2. Integrating community and policy changes should help make mental health services move towards de-formalization. This process increases the chance of the patient being returned to the community and living in an environment free from coercion.  3. Need a real implementation that ensure patients live in their families and communities free from coercion. |
