# Supplementary material for: The use of pasung for people with mental illness: a systematic review and narrative synthesis
Source: Int J Ment Health Syst. 2020 Dec 7;14:90. doi: 10.1186/s13033-020-00424-0 (PMC7720453; doi:10.1186/s13033-020-00424-0)
Supplement: Supplementary file 3 — Additional file 3. Additional tables. [file 13033_2020_424_MOESM3_ESM.docx]

**Additional File: Quality Ratings**

**Table S3: CASP – Qualitative Studies**

| Question number | Article/ question | Asher, et al., 2017 [7] | | | Broch, 2001 [6] | | | Buanasari, et al., 2018 [29] | | | Hall, et al., 2019 [35] | | | Irmansyah, et al., 2009 [8] | | | Katuuk, Daulima & Wardani, 2019 [9] | | | Laila, et al., 2018 [40] | | | Nurjannah, et al., 2015 [16] | | | Helena, Daulima & Wardani, 2018 [49] | | | Read, Adiibokah & Nyame, 2009 [4] | | | Riany, et al., 2016 [51] | | |
| --- | --- | --- | --- | --- | --- | --- | --- | --- | --- | --- | --- | --- | --- | --- | --- | --- | --- | --- | --- | --- | --- | --- | --- | --- | --- | --- | --- | --- | --- | --- | --- | --- | --- | --- |
| 1 | Was there a clear statement of the aims of the research? | Y |  |  | Y |  |  | Y |  |  | Y |  |  | Y |  |  | Y |  |  | Y |  |  | Y |  |  | Y |  |  | Y |  |  | Y |  |  |
| 2 | Is a qualitative methodology appropriate? | Y |  |  | Y |  |  | Y |  |  | Y |  |  | Y |  |  | Y |  |  | Y |  |  | Y |  |  | Y |  |  | Y |  |  | Y |  |  |
| 3 | Was the research design appropriate to address the aims of the research? | Y |  |  | Y |  |  | Y |  |  | Y |  |  |  |  | ? | Y |  |  | Y |  |  | Y |  |  | Y |  |  | Y |  |  | Y |  |  |
| 4 | Was the recruitment strategy appropriate to the aims of the research? | Y |  |  | Y |  |  | Y |  |  | Y |  |  |  |  | ? | Y |  |  | Y |  |  | Y |  |  |  |  | ? | Y |  |  | Y |  |  |
| 5 | Was the data collected in a way that addressed the research issue? | Y |  |  | Y |  |  | Y |  |  | Y |  |  |  |  | ? |  |  | ? | Y |  |  | Y |  |  |  |  | ? | Y |  |  | Y |  |  |
| 6 | Has the relationship between researcher and participant been adequately considered? |  |  | ? | Y |  |  |  |  | ? |  |  | ? |  |  | ? |  |  | ? |  |  | ? |  |  | ? |  |  | ? | Y |  |  |  |  | ? |
| 7 | Have ethical issues been taken into consideration? | Y |  |  |  | N |  | Y |  |  |  |  | ? |  |  | ? | Y |  |  | Y |  |  | Y |  |  | Y |  |  | Y |  |  | Y |  |  |
| 8 | Was the data analysis sufficiently rigorous? |  |  | ? | Y |  |  |  |  | ? |  |  | ? |  |  | ? |  |  | ? | Y |  |  | Y |  |  |  |  | ? | Y |  |  | Y |  |  |
| 9 | Is there a clear statement of the findings? | Y |  |  | Y |  |  | Y |  |  | Y |  |  | Y |  |  | Y |  |  | Y |  |  | Y |  |  | Y |  |  | Y |  |  | Y |  |  |
| 10 | How valuable is the research? | Y |  |  | Y |  |  | Y |  |  | Y |  |  | Y |  |  | Y |  |  | Y |  |  | Y |  |  |  |  | ? | Y |  |  | Y |  |  |
|  | **Total CASP Score** | 8 |  |  | 9 |  |  | 8 |  |  | 7 |  |  | 4 |  |  | 7 |  |  | 9 |  |  | 9 |  |  | 5 |  |  | 10 |  |  | 9 |  |  |

| Question number | Article/ question | Tanaka, et al., 2018 [57] | | | Tay, et al., 2017 [58] | | | Wulandari, Daulima & Wardani, 2019 [62] | | | Daulima, Rasmawati & Wardani, 2019 [30] | | | Firdaus, 2007 [34] | | | Rahman, Marchira & Rahmat, 2016 [48] | | | | Nova Helena, 2018 [49] | | | Reknoningsih, Daulima & Putri, 2014 [50] | | | Wirya, 2014 [61] | | | Yusuf & Tristiana, 2018 [10] | | |
| --- | --- | --- | --- | --- | --- | --- | --- | --- | --- | --- | --- | --- | --- | --- | --- | --- | --- | --- | --- | --- | --- | --- | --- | --- | --- | --- | --- | --- | --- | --- | --- | --- |
| 1 | Was there a clear statement of the aims of the research? | Y |  |  | Y |  |  |  |  | ? | Y |  |  |  |  | ? | | Y |  |  | Y |  |  | Y |  |  | Y |  |  | Y |  |  |
| 2 | Is a qualitative methodology appropriate? | Y |  |  | Y |  |  | Y |  |  | Y |  |  |  |  | ? | | Y |  |  | Y |  |  | Y |  |  | Y |  |  | Y |  |  |
| 3 | Was the research design appropriate to address the aims of the research? | Y |  |  | Y |  |  |  |  | ? | Y |  |  |  |  | ? | | Y |  |  | Y |  |  |  |  | ? | Y |  |  | Y |  |  |
| 4 | Was the recruitment strategy appropriate to the aims of the research? | Y |  |  | Y |  |  |  |  | ? |  |  | ? |  |  | ? | | Y |  |  | Y |  |  |  |  | ? | Y |  |  | Y |  |  |
| 5 | Was the data collected in a way that addressed the research issue? | Y |  |  | Y |  |  |  |  | ? | Y |  |  |  |  | ? | |  |  | ? |  |  | ? |  |  | ? | Y |  |  | Y |  |  |
| 6 | Has the relationship between researcher and participant been adequately considered? |  |  | ? |  |  | ? |  |  | ? |  |  | ? |  | N |  | |  |  | ? |  |  | ? |  | N |  |  | N |  |  |  | ? |
| 7 | Have ethical issues been taken into consideration? | Y |  |  |  | N |  | Y |  |  | Y |  |  |  |  | ? | |  |  | ? |  |  | ? |  | N |  |  |  | ? |  |  | ? |
| 8 | Was the data analysis sufficiently rigorous? | Y |  |  |  |  | ? |  |  | ? | Y |  |  |  | N |  | | Y |  |  | Y |  |  |  | N |  |  |  | ? | Y |  |  |
| 9 | Is there a clear statement of the findings? | Y |  |  |  |  | ? |  |  | ? |  |  | ? |  |  | ? | |  | N |  |  | N |  |  |  | ? |  |  | ? |  |  | ? |
| 10 | How valuable is the research? | Y |  |  | Y |  |  |  |  | ? | Y |  |  |  |  | ? | |  |  | ? | Y |  |  |  |  | ? | Y |  |  | Y |  |  |
|  | **Total CASP Score** | 9 |  |  | 6 |  |  | 2 |  |  | 7 |  |  | 0 |  |  | | 5 |  |  | 6 |  |  | 2 |  |  | 6 |  |  | 7 |  |  |

**Table S4: CASP - Surveys**

| Question number | Article/ question | Daulima, 2018 [31] | | | Hartini, et al., 2018 [36] | | | Puteh, et al., 2011 [47] | | | Suhron, Yusuf & Subarniati, 2018 [56] | | | Vijayalakshmi, et al., 2012 [60] | | | Idaiani & Raflizar, 2017 [38] | | | Suharto, 2014 [54] | | |
| --- | --- | --- | --- | --- | --- | --- | --- | --- | --- | --- | --- | --- | --- | --- | --- | --- | --- | --- | --- | --- | --- | --- |
| 1 | Did the study address a clearly focused question / issue? | Y |  |  | Y |  |  | Y |  |  |  |  | ? | Y |  |  | Y | N | ? | Y | N | ? |
| 2 | Is the research method (study design) appropriate for answering the research question? | Y |  |  | Y |  |  | Y |  |  |  |  | ? | Y |  |  | Y | N | ? | Y | N | ? |
| 3 | Is the method of selection of the subjects (employees, teams, divisions, organizations) clearly described? | Y |  |  | Y |  |  | Y |  |  |  | N |  | Y |  |  | Y | N | ? | Y | N | ? |
| 4 | Could the way the sample was obtained introduce (selection)bias? | Y |  |  | Y |  |  |  | N |  | Y |  |  |  | N |  | Y | N | ? | Y | N | ? |
| 5 | Was the sample of subjects representative with regard to the population to which the findings will be referred? | Y |  |  | Y |  |  | Y |  |  |  |  | ? | Y |  |  | Y | N | ? | Y | N | ? |
| 6 | Was the sample size based on pre-study considerations of statistical power? | Y |  |  |  | N |  |  | N |  |  | N |  |  |  | ? | Y | N | ? | Y | N | ? |
| 7 | Was a satisfactory response rate achieved? | Y |  |  | Y |  |  | Y |  |  | Y |  |  | Y |  |  | Y | N | ? | Y | N | ? |
| 8 | Are the measurements (questionnaires) likely to be valid and reliable? | Y |  |  |  |  | ? |  |  | ? |  |  | ? | Y |  |  | Y | N | ? | Y | N | ? |
| 9 | Was the statistical significance assessed? | Y |  |  | Y |  |  | Y |  |  | Y |  |  | Y |  |  | Y | N | ? | Y | N | ? |
| 10 | Are confidence intervals given for the main results? |  | N |  |  | N |  |  | N |  |  | N |  |  |  | ? | Y | N | ? | Y | N | ? |
| 11 | Could there be confounding factors that haven’t been accounted for? |  |  | ? | Y |  |  | Y |  |  | Y |  |  |  | N | ? | Y | N | ? | Y | N | ? |
| 12 | Can the results be applied to your organization? (your setting/context) # |  |  |  |  |  |  |  |  |  |  |  |  |  |  |  |  |  |  |  |  |  |
|  | **Total CASP Score** | 9 |  |  | 8 |  |  | 7 |  |  | 4 |  |  | 7 |  |  | 11 |  |  | 11 |  |  |

# - Not applicable

**Table S5: JBI – Before-After Studies**

| Question number | Article/ question | Dewi, et al., 2019 [19] | | | Guan, et al., 2015 [2] | | | Suhron, 2017 [55] | | | Suryani, et al., 2011 [5] | | |
| --- | --- | --- | --- | --- | --- | --- | --- | --- | --- | --- | --- | --- | --- |
| 1 | Is it clear in the study what is the 'cause' and what is the 'effect'? |  | N |  | Y |  |  | Y |  |  | Y |  |  |
| 2 | Were the participants included in any comparisons similar? |  |  | ? |  |  | ? |  |  | ? |  |  | ? |
| 3 | Were the participants included in any comparisons receiving similar treatment/care, other than the exposure or intervention of interest? |  |  | ? |  |  | ? |  |  | ? |  |  | ? |
| 4 | Was there a control group? | Y |  |  |  | N |  |  | N |  |  | N |  |
| 5 | Were there multiple measurements of the outcome both pre and post the interventions/exposure? | Y |  |  | Y |  |  | Y |  |  |  |  | ? |
| 6 | Was follow-up complete, and if not, was follow-up adequately reported and strategies to deal with loss to follow-up employed? |  |  | ? |  | N |  |  |  | ? |  |  | ? |
| 7 | Were the outcomes of participants included in any comparisons measured in the same way? |  |  | ? |  |  | ? |  |  | ? |  |  | ? |
| 8 | Were outcomes measured in a reliable way? | Y |  |  | Y |  |  |  |  | ? |  |  | ? |
| 9 | Was appropriate statistical analysis used? |  |  | ? | Y |  |  |  |  | ? |  |  | ? |
|  | **Total JBI Score** | 3 |  |  | 4 |  |  | 2 |  |  | 1 |  |  |

**Table S6: JBI – Cross Sectional Studies**

| Question number | Article/ question | Minas & Diatri, 2008 [3] | | |
| --- | --- | --- | --- | --- |
| 1 | Were the criteria for inclusion in the sample clearly defined? |  |  | N |
| 2 | Were the study subjects and the setting described in detail? |  | N |  |
| 3 | Was the exposure measured in a valid and realiable way? | Y |  |  |
| 4 | Were objective, standardised criteria used for measurement of the condition? | Y |  |  |
| 5 | Were confounding factors identified? |  | N |  |
| 6 | Were strategies to deal with confounding factors stated? |  | N |  |
| 7 | Were the outcomes measured in a valid and reliable way? | Y |  |  |
| 8 | Was appropriate statistical analysis used? |  |  | ? |
|  | **Total JBI Score** | 3 |  |  |

**Table S7: JBI – Case Controlled Studies**

| Question number | Article/ question | Laila, et al., 2019 [41] | | |
| --- | --- | --- | --- | --- |
| 1 | Did the study address a clealy focused issue? | Y |  |  |
| 2 | Did the authors use an appropiate method to answer their question? | Y |  |  |
| 3 | Were the cases recruited in an acceptable way? | Y |  |  |
| 4 | Were the controls selected in a acceptable way? | Y |  |  |
| 5 | Was the exposure accurately measured to minimise the bias? | Y |  |  |
| 6a | Aside from the experimental intervention, were the gropus treted equally? | Y |  |  |
| 6b | Have the authors taken account of the potential confounding factors in the design and/or in their analysis? |  |  | ? |
| 7 | How large was the treatment effect? |  |  | ? |
| 9 | How precise was the estimate of the treatmnet effect? | Y |  |  |
| 9 | Do you believe the results? | Y |  |  |
| 10 | Can the results be applied to the local population? | Y |  |  |
| 11 | Do the results of this study fit with other available evidence? | Y |  |  |
|  | **Total JBI Score** | 10 |  |  |
